# Microbial network inference for longitudinal microbiome studies with LUPINE

**DOI:** 10.1186/s40168-025-02041-w

**Published:** 2025-03-03

**Authors:** Saritha Kodikara, Kim-Anh Lê Cao

**Affiliations:** Melbourne Integrative Genomics, School of Mathematics and Statistics, The University of Melbourne, Royal Parade, 3052 Parkville, Victoria Australia

**Keywords:** Longitudinal, Network, 16S, Partial correlation

## Abstract

**Background:**

The microbiome is a complex ecosystem of interdependent taxa that has traditionally been studied through cross-sectional studies. However, longitudinal microbiome studies are becoming increasingly popular. These studies enable researchers to infer taxa associations towards the understanding of coexistence, competition, and collaboration between microbes across time. Traditional metrics for association analysis, such as correlation, are limited due to the data characteristics of microbiome data (sparse, compositional, multivariate). Several network inference methods have been proposed, but have been largely unexplored in a longitudinal setting.

**Results:**

We introduce LUPINE (LongitUdinal modelling with Partial least squares regression for NEtwork inference), a novel approach that leverages on conditional independence and low-dimensional data representation. This method is specifically designed to handle scenarios with small sample sizes and small number of time points. LUPINE is the first method of its kind to infer microbial networks across time, while considering information from all past time points and is thus able to capture dynamic microbial interactions that evolve over time. We validate LUPINE and its variant, LUPINE_single (for single time point analysis) in simulated data and four case studies, where we highlight LUPINE’s ability to identify relevant taxa in each study context, across different experimental designs (mouse and human studies, with or without interventions, and short or long time courses). To detect changes in the networks across time and groups or in response to external disturbances, we used different metrics to compare the inferred networks.

**Conclusions:**

LUPINE is a simple yet innovative network inference methodology that is suitable for, but not limited to, analysing longitudinal microbiome data. The R code and data are publicly available for readers interested in applying these new methods to their studies.

Video Abstract

**Supplementary Information:**

The online version contains supplementary material available at 10.1186/s40168-025-02041-w.

## Introduction

The microbiome refers to a distinct microbial community that resides within a well-defined habitat, characterised by specific physio-chemical properties [1]. This ecosystem is composed of bacteria from hundreds to thousands of different taxa [2], forming complex community structures that are challenging to decipher. Given the inherent dynamics of the microbiome, longitudinal studies are essential to gain insights into the impact, variations over time, and responses to external perturbations such as dietary changes or medications on the microbiome. The temporal dimension, while adding complexity, provides richer insights into the dynamic processes, patterns, and interactions within microbial communities, surpassing what cross-sectional studies can offer [3]. The advancements in sequencing technologies, coupled with reduced sequencing costs, have enabled researchers to conduct an increasing number of longitudinal microbiome studies.

One objective of microbiome studies is to accurately infer microbial networks from the datasets generated by sequencing technologies. These networks can be viewed as temporal or spatial snapshots of ecosystems [4]. In these networks, interactions signify significant associations between taxa, which are typically non-directional in nature [5]. The simplest way to detect associations is via correlation analysis, such as Pearson or Spearman correlation. However, these correlation methods are sub-optimal for microbiome data as they ignore the compositional structure of the data and lead to spurious results [6]. A more valid approach is to use the concept of partial correlation, which effectively measure pairwise associations between variables subject to a constant-sum constraint (i.e. compositional data, [7]). Existing compositional-aware microbiome network methods have currently focused on single time point studies, rather than longitudinal. Widely used single time point methods are either based on correlation approaches, such as SparCC [8] or precision based approaches, such as SpiecEasi [5]. In contrast to correlation-based methods, precision or partial correlation-based methods concentrate on direct associations by removing indirect associations.

Longitudinal microbiome studies enables researchers to create temporal snapshots of microbial networks for a comprehensive view of the microbiome. But network methods for such type of studies are still in their infancy and exhibit diverse analytical objectives [9]. Some methods aim to model relationships between taxa for each individual subject. Other methods aim to model collective microbial interactions across all time points. However, the assumption that microbial interaction stays constant through time, whether on an individual basis or across all subjects, proves limiting. This limitation is particularly evident when there are external interventions, such as alterations in diet or antibiotic usage, which disrupt the stability of these relationships. We have addressed this limitation with a sequential approach, that, to the best of our knowledge, is the first of its kind for longitudinal microbiome studies. We acknowledge the dynamic nature of microbial interactions, especially in the face of interventions, and introduce a flexible framework for the temporal evolution of these interactions.

LUPINE (LongitUdinal modelling with Partial least squares regression for NEtwork inference) combines one-dimensional approximation and partial correlation to measure the linear association between a pair of taxa, accounting for the effects of the other taxa. We developed two variants, one for single time point network inference, and another for longitudinal sequential network inference. The former focuses on inferring the network at a specific time point, in the vein of SpiecEasi [5] and SparCC [8]. The latter, however, incorporates information from all previous time points. The inferred network from our method is in the format of a binary graph. In this network, the vertices represent different taxa, and the edges represent significant associations between them. An edge between two taxa indicates that there is a significant connection between them. This connection is visualised as a line between the two nodes in the graph.

In the ‘Materials and methods’ section, we explain the rationale of our approaches along with downstream analyses that can be conducted to compare inferred networks. In the ‘Results’ section, we first benchmark LUPINE and LUPINE_single with two state-of-the-art network inference methods (solely designed for single time point microbiome data) on simulated data. Next, through a case study, we demonstrate the robustness of LUPINE compared to LUPINE_single. We then showcase the applicability of LUPINE in real data with different experimental designs in four case studies. These experiments varied from human studies to mouse studies, from short to longer time courses, and ranged from cross-sectional to intervention studies. Finally, in the ‘Discussion’ section, we delve into the implications of our findings, special aspects we had to consider when developing LUPINE, and suggest avenues for future research.

## Materials and methods

### Method

Our network inference methodology is tailored for the analysis of longitudinal microbiome data, where we assume that the dynamics of taxa interactions are temporal. In order to uncover these intricate relationships, we leverage both past and current information from the data in a sequential manner. Including past information in the model enables insights into inherent variations in microbial interactions across diverse temporal scenarios, including interventions. Our methodology includes three variants that are described in the following sections:

- Single time point modelling: given a pair of taxa, we estimate pairwise partial correlations while accounting for the influence of the other taxa using one-dimensional approximation with principal component analysis (PCA).
- Two time point modelling: given a pair of taxa, we estimate pairwise partial correlations while accounting for the influence of the other taxa using projection to latent structures (PLS) regression [10]. PLS regression performs dimension reduction such that the covariance between the current and preceding time point data sets is maximised.
- Several time point modelling: given a pair of taxa, we estimate pairwise partial correlations while accounting for the influence of the other taxa using generalised PLS for multiple blocks of data (‘blockPLS’) regression [11, 12]. We use blockPLS to maximise the covariance between the current and any past time point datasets.

We assume that individuals within a specific group (e.g. control group) have a common network structure at a particular time point. Therefore, we consider each group separately for studies with multiple sample groups (as illustrated in case studies 1, 3 and 4) to focus on each of their unique microbial characteristics.

#### Notations.

In the following, we denote $\varvec{X}$ an ($n \times p$) data matrix (where *n* is the number of samples and *p* is the number of taxa); $\varvec{X}^i$ and $\varvec{X}^j$ are columns in $\varvec{X}$ for taxa *i* and *j*; $\varvec{X}^{-(i,j)}$ is an ($n \times p-2$) data matrix excluding taxa *i* and *j* columns in ***X***; ***l*** is a vector of library sizes for *n* samples. In situations where time is of interest (i.e. in longitudinal modelling), all of the above matrices and vectors include a subscript indicating the time point. For example, $\varvec{X}_t$ represents an ($n \times p$) data matrix at time point *t*. For simplicity, we will omit the hat notation (i.e. $\hat{\rho }$) of our estimates. However, note that all estimates approximate some population parameter. Additionally, $\Vert .\Vert$ denotes the Euclidean norm.

### Single time point modelling with PCA

We first introduce the single time point approach which we will generalise for multiple time points in the next sections. This type of analysis gives insights of microbial associations at a single time point. It can also be used when there is a large time gap between time points but only one particular time point is of interest.

**Fig. 1:**
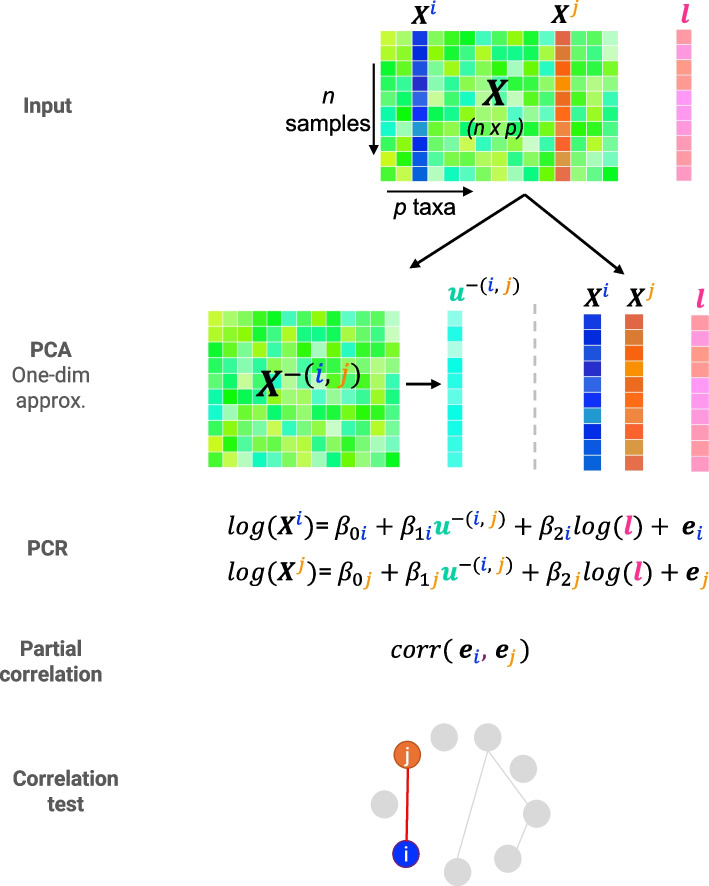
Single time point modelling overview. Consider the ($n \times p$) input data matrix ***X*** and ***l*** the library size of each sample. First, we use PCA to derive a one-dimensional approximation, $\varvec{u}^{-(i,j)}$ to control for taxa other than *i* and *j* from the $\varvec{X}$ data matrix (i.e. ${X}^{-(i,j)}$). We then apply two independent log linear regression models (a.k.a. principal component regression) for $\varvec{X}^i$ and $\varvec{X}^j$, regressed on component $\varvec{u}^{-(i,j)}$ and $\log (\varvec{l})$ to obtain the residuals $\varvec{e}_i$ and $\varvec{e}_j$. We estimate the partial correlation estimate for taxa *i* and *j* as the correlation between the two residuals. Finally, we test for the statistical significance of the true correlation between $\varvec{e}_i$ and $\varvec{e}_j$ to infer whether taxa *i* and *j* are connected in the network. We iterate this process for all pairs of taxa (*i*, *j*) to obtain a full binary network. The ‘Improvement in computational time’ section explains how to avoid repeated PCA approximations to improve computational time

To assess the strength of the association between two taxa, we use partial correlation―a statistical technique that measures association while controlling for other variables. For example, we estimate the partial correlation between a pair of taxa, denoted by *i* and *j* in Fig. 1, to assess the strength of their association while controlling for other taxa. Controlling for these taxa requires regressing taxa *i* and *j* against $p-2$ taxa, which becomes unsolvable when the number of taxa *p* is larger than the number of samples *n* (e.g. in typical microbiome studies $p = 150 - 500$ and $n< 50$). Hence, the first step in our approach is to calculate a one-dimensional approximation of the control variables (e.g. all taxa except the pair *i* and *j*), through the first principal component. Using simulations (see the ‘Effect of using different number of components in the deflation’ section in Appendix D), we demonstrate that using a single component produces more accurate network inference than using two or three components to approximate the control variables specifically when sample sizes are small. However, this may not be true for all datasets. Therefore, in our methods (i.e. LUPINE_single and LUPINE), we encourage users to explore different numbers of components when approximating the control variables in the model.

We first start with a generic formulation of PCA [13]. The objective function to obtain the first principal component is

1 $$\begin{aligned} \underset{\Vert {\varvec{w}}\Vert =1}{\text {argmax}}\ {var(\varvec{wX})} \end{aligned}$$

where ***w*** is the *p* dimensional loading vector associated to the first principal component ***u***, with $\varvec{u}=\varvec{wX}$ under the constraint that *w* is of unit (norm) 1.

First, we modify Eq. (1) to find the first principal component of the control taxa that excludes taxa *i* and *j* as

2 $$\begin{aligned} \underset{\Vert {\varvec{w}^{-(i,j)}}\Vert =1}{\text {argmax}} {var\left( \varvec{w}^{-(i,j)}\varvec{X}^{-(i,j)}\right) } \end{aligned}$$

where $\varvec{w}^{-(i,j)}$ is the $(p-2)$ dimensional loading vector associated to the first principal component $\varvec{u}^{-(i,j)}$ with $\varvec{u}^{-(i,j)}=\varvec{w}^{-(i,j)}\varvec{X}^{-(i,j)}$.

Next, we fit two log linear regressions on each of the taxa *i* and *j*. They are regressed against the first principal component, $\varvec{u}^{-(i,j)}$, similar to principal component regression (PCR, [14]). PCR combines PCA and multiple linear regression, where the principal components of the explanatory variables serve as predictors rather than using the original variables directly in the regression. Here, we fit two log linear regressions to extract their residuals denoted by $\varvec{e}_i$ and $\varvec{e}_j$:

3 $$\begin{aligned} \varvec{e}_i= log\left(\varvec{X}^i\right) - E\left(log\left(\varvec{X}^i\right)|\varvec{u}^{-(i,j)}\right); \qquad \varvec{e}_j= log\left(\varvec{X}^j\right) - E \left(log\left(\varvec{X}^j\right)|\varvec{u}^{-(i,j)}\right) \end{aligned}$$

where $E\left(.|\varvec{u}^{-(i,j)}\right)$ is the conditional expectation with respect to the first principal component $\varvec{u}^{-(i,j)}$. We use a log linear regression as the response variables are counts. This is similar to the bias-corrected version of ANCOM [15] that uses a linear regression framework based on log-transformed taxa counts. We add an offset of 1 to zero counts to ensure that the log transformation is valid. Additionally, in the ‘Effect of normalisation’ section in Appendix D, we assess the impact of using log-transformed counts in Eq. 2.

In addition, to correct for library size (i.e. the variation in the total sequence reads), we modify Eq. 3 and include an offset, similar to the approach of [16, 17]:

4 $$\begin{aligned} \varvec{e}_i= log\left(\varvec{X}^i\right) - E\left(log\left(\varvec{X}^i\right)|\varvec{u}^{-(i,j)}, log(\varvec{l})\right); \qquad \varvec{e}_j= log\left(\varvec{X}^j\right) - E\left(log\left(\varvec{X}^j\right)|\varvec{u}^{-(i,j)}, log(\varvec{l})\right) \end{aligned}$$

where $E\left(.|\varvec{u}^{-(i,j)}, log(\varvec{l})\right)$ is the conditional expectation with respect to the first principal component $\varvec{u}^{-(i,j)}$ and the log library size $\log (\varvec{l})$.

We then quantify the strength of association between taxa *i* and *j* based on partial correlation using the residuals $\varvec{e}_i$ and $\varvec{e}_j$. Partial correlations reveal direct (i.e. conditionally independent) associations between two variables while controlling for the other variables. We calculate the partial correlation between the pair of taxa *i* and *j* as

5 $$\begin{aligned} \pi _{\varvec{e}_i,\varvec{e}_j}= corr(\varvec{e}_i,\varvec{e}_j). \end{aligned}$$

Finally, we test whether the true correlation $\rho _{\varvec{e}_i,\varvec{e}_j}$ between $\varvec{e}_i$ and $\varvec{e}_j$ (estimated with $\pi _{\varvec{e}_i,\varvec{e}_j}$) is significantly different from zero. The correlation test assumes that when the correlation is zero, the test statistic $t= \pi _{\varvec{e}_i,\varvec{e}_j} \sqrt{\frac{n-2}{1-\pi _{\varvec{e}_i,\varvec{e}_j}^2}}$, follows a normal distribution. Thus, we test the hypothesis $H_0: \rho _{\varvec{e}_i,\varvec{e}_j} = 0$ versus $H_1: \rho _{\varvec{e}_i,\varvec{e}_j} \ne 0$ using a t-distribution with $n-2$ degrees of freedom. We then consider that a pair of taxa is connected in a network if the associated *p*-value from the correlation test is less than a chosen significance level (e.g. 0.05). We apply this approach to all possible pairs of taxa to obtain a binary network summarising the pairwise connections between all taxa. In the ‘Permutation test vs. Correlation test’ section in Appendix D, we compare this analytical approach with a permutation-based *p*-value calculation.

To quantify the uncertainty of the correlation test *p*-value, we use bootstrapping, which helps determine the significance of a correlation. We resample the data set 1000 times and calculate the *p*-values from correlation tests for each iteration. This process generates a confidence interval for each *p*-value, allowing us to better assess the significance of the correlation. We demonstrate this approach in case study 4.

In the pseudo-algorithm 1 and Fig. 1, we outline the different steps in LUPINE_single. In the Improvement in computational time section, we explain how we improve computational time by avoiding repeated PCA approximations.

**Figure:**
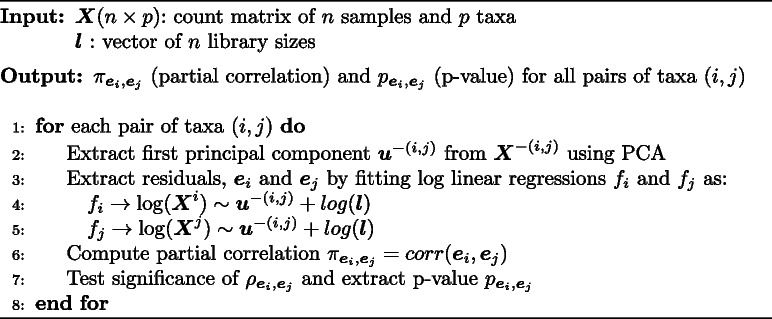
**Algorithm 1** Single time point scheme

### Two time point modelling with PLS regression

**Fig. 2:**
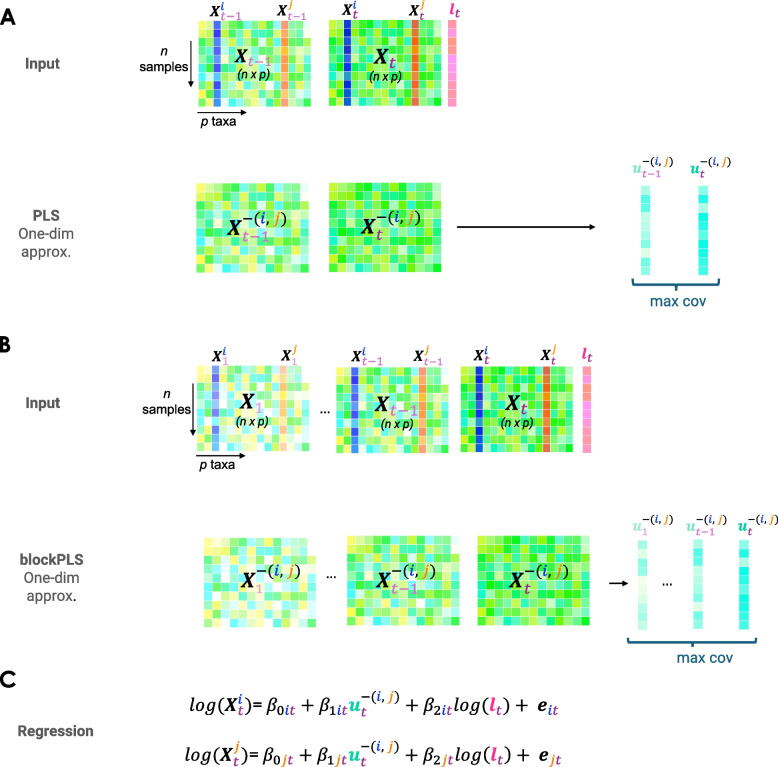
Two or more time points modelling overview. Consider as input the ($n \times p$) data matrices for **A** two time points or **B** more than two time points (denoted $\varvec{X}_{1} \dots , {\textbf {X}}_{t}$) and their respective library size $\varvec{l}_t$ at time *t*. Similar to single time point modelling presented in Fig. 1, first, we derive a one-dimensional approximation, $\varvec{u}^{-(i,j)}_t$ to control for taxa other than *i* and *j*. Instead of using PCA as previously for a single time point, we use either **A** PLS maximising the covariance with the control taxa at the preceding time $t-1$, or **B** blockPLS maximising the covariance with the control taxa at all previous times $1,\ldots ,t-1$. **C** We then fit two independent log linear models for $\varvec{X}^i_t$ and $\varvec{X}^j_t$, regressed on $\varvec{u}^{-(i,j)}_t$ and $\log (\varvec{l}_t)$ to compute the residuals $\varvec{e}_{it}$ and $\varvec{e}_{jt}$ following either **A** or **B**. The remainder of the modelling process, including the partial correlation calculation and the correlation test, is identical to that in Fig. 1

Here, we use PLS to obtain a one-dimensional approximation between two datasets from two time points (Fig. 2). PLS is a projection-based method that maximises the covariance between the latent components associated to two data sets while managing correlated information [10]. The PLS latent variables (or components), are linear combinations of variables from each data set. These latent variables aim to uncover subtle biological effects not evident otherwise if each data set is considered independently [12].

The objective function for the first dimension of PLS is

6 $$\begin{aligned} \underset{\Vert {\varvec{w}_{t-1}}\Vert =1, \Vert {\varvec{w}_t}\Vert =1}{\text {argmax}} {Cov\left(\varvec{w}_{t-1}\varvec{X}_{t-1}, \varvec{w}_t\varvec{X}_t\right)} \end{aligned}$$

where $\varvec{w}_{t-1}$ and $\varvec{w}_t$ are the two *p* dimensional loading vectors associated to the first latent components $\varvec{u}_{t-1}=\varvec{w}_{t-1}\varvec{X}_{t-1}$ and $\varvec{u}_t=\varvec{w}_t\varvec{X}_t$.

We modify Eq. 6 to find the first latent components of the controlling taxa at time point *t* that has a maximum covariance with the preceding time point latent component as

7 $$\begin{aligned} \underset{\Vert {\varvec{w}^{-(i,j)}_{t-1}}\Vert =1, \Vert {\varvec{w}^{-(i,j)}_t}\Vert =1}{\text {argmax}} {Cov\left(\varvec{w}^{-(i,j)}_{t-1}\varvec{X}^{-(i,j)}_{t-1}, \varvec{w}^{-(i,j)}_t\varvec{X}^{-(i,j)}_t\right)} \end{aligned}$$

where $\varvec{w}^{-(i,j)}_{t-1}$ and $\varvec{w}^{-(i,j)}_t$ are the two $(p-2)$ loading vectors associated to the first latent components $\varvec{u}^{-(i,j)}_{t-1}=\varvec{w}_{t-1}\varvec{X}^{-(i,j)}_{t-1}$ and $\varvec{u}^{-(i,j)}_t=\varvec{w}_t\varvec{X}^{-(i,j)}_t$.

To infer the network at time point *t* using the information from the preceding time point ($t-1$), we fit two log linear regressions. These regressions are fitted on $\varvec{X}^i_t$ and $\varvec{X}^j_t$ that are regressed against the first latent component $\varvec{u}^{-(i,j)}_t$ and the log library size $\log (\varvec{l}_t)$. Note that $\varvec{u}^{-(i,j)}_t$ differs from the single time point approach $\varvec{u}^{-(i,j)}$ as we maximise the covariance with the preceding time point latent component $\varvec{u}^{-(i,j)}_{t-1}$. The updated Eqs. 4 and 5 become

8 $$\begin{aligned} \varvec{e}_{it}= log\left(\varvec{X}^i_t\right) - E\left(log\left(\varvec{X}^i_t\right)|\varvec{u}^{-(i,j)}_t, log\left(\varvec{l}_t\right)\right); \qquad \varvec{e}_{jt}= log\left(\varvec{X}^j_t\right) - E\left(log\left(\varvec{X}^j_t\right)|\varvec{u}^{-(i,j)}_t, log(\varvec{l}_t)\right) \end{aligned}$$

9 $$\begin{aligned} \pi _{\varvec{e}_{it},\varvec{e}_{jt}}= corr(\varvec{e}_{it},\varvec{e}_{jt}) \end{aligned}$$

where $E\left(.|\varvec{u}^{-(i,j)}_t, log(\varvec{l}_t)\right)$ is the conditional expectation with respect to the first latent component $\varvec{u}^{-(i,j)}_t$ and the log library size, $\log (\varvec{l}_t)$ at time point *t*; and $\varvec{e}_{it}$ and $\varvec{e}_{jt}$ are the residuals from the two log linear models.

Similar to the single time point scheme described above, we test for the strength of association between taxa *i* and *j* with a correlation test and obtain a binary network summarising the pairwise connections between all taxa after controlling for all other taxa from current time point and previous time point.

### Multiple time point modelling with blockPLS regression

We use blockPLS, an extension of PLS, to calculate a one-dimensional approximation that maximises the covariance between latent components associated to the current time point and all past time points. BlockPLS is based on generalised PLS [11, 12] that involves regressing the response matrix (e.g. $\varvec{X}_t$) on multiple datasets ($\varvec{X}_1, \varvec{X}_2, \ldots , \varvec{X}_{t-1}$).

The objective function for the first dimension of blockPLS is

10 $$\begin{aligned} \underset{\Vert {\varvec{w}_1}\Vert =1, \ldots , \Vert {\varvec{w}_t}\Vert =1}{\text {argmax}} \sum\limits_{q,k=1, q\ne k}^{t}{Cov\left(\varvec{w}_q\varvec{X}_q, \varvec{w}_k\varvec{X}_k\right)} \end{aligned}$$

where $\varvec{w}_q$ is a *p* dimensional loading vector associated to the first latent component $\varvec{u}_q$, where $\varvec{u}_q=\varvec{w}_q\varvec{X}_q$.

We modify Eq. 10 to find the first latent components of all controlling taxa at time point *t* that has a maximum covariance with previous time points latent components as

11 $$\begin{aligned} \underset{\Vert {\varvec{w}^{-(i,j)}_1}\Vert =1, \ldots , \Vert {\varvec{w}^{-(i,j)}_t}\Vert =1}{\text {argmax}} \sum _{q,k=1, q\ne k}^{t}{Cov\left(\varvec{w}^{-(i,j)}_q\varvec{X}^{-(i,j)}_q, \varvec{w}^{-(i,j)}_k\varvec{X}^{-(i,j)}_k\right)} \end{aligned}$$

where $\varvec{w}^{-(i,j)}_q$ is a $p-2$ dimensional loading vector associated to the first latent component $\varvec{u}^{-(i,j)}_q$ at time point *q*, with $\varvec{u}^{-(i,j)}_q=\varvec{w}^{-(i,j)}_q\varvec{X}^{-(i,j)}_q$.

To infer a network at any time point *t* using all time points until time point *t*, we fit two log linear regressions on the counts of $\varvec{X}^i_t$ and $\varvec{X}^j_t$ against the first latent component at *t*, $\varvec{u}^{-(i,j)}_t$, and log library size at *t*, $\log (\varvec{l}_t)$. However, we need to keep in mind that the latent component $\varvec{u}^{-(i,j)}_t$ maximises the covariance with all previous time points latent variables, $\left[\varvec{u}^{-(i,j)}_1, \ldots , \varvec{u}^{-(i,j)}_{t-1}\right]$, thus effectively taking into account all past time points. We then use Eqs. 8 and 9 to quantify and test the statistical significance of the partial correlation between taxa *i* and *j*.

Pseudo-algorithm 2 outlines the different steps in either two time points and multiple time points modelling.

**Figure:**
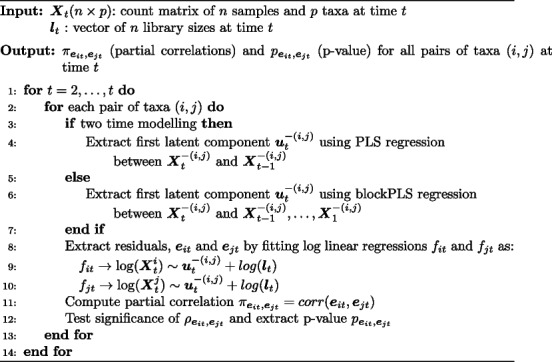
**Algorithm 2** Multi time point scheme

Note that in both PCA and PLS versions we centre and scale each taxon to avoid scaling issues.

### Improvement in computational time

Our approach requires to compute one-dimensional approximation for each pair of taxa (*i*, *j*), i.e. $p*(p-1)/2$ times. Using matrix decomposition principles, we propose instead to approximate the full data matrix ***X*** with the first one-dimension component ***u*** that summarises most of the variation in ***X***, and its associated loading vector that are obtained from PCA, PLS, or blockPLS:

12 $$\begin{aligned} \left[ \begin{array}{cccc} x_{11} & x_{12} & \cdots & x_{1p}\\ x_{21} & x_{22} & \cdots & x_{2p}\\ \vdots & \vdots & \ddots & \vdots \\ x_{n1} & x_{n2} & \cdots & x_{np} \end{array}\right] _{n \times p} \approx \left[ \begin{array}{c} u_{1} \\ u_{2} \\ \vdots \\ u_{n} \end{array}\right] _{n \times 1} \times \left[ \begin{array}{cccc} w_{1}&w_{2}&\cdots&w_{p} \end{array}\right] _{1 \times p} \end{aligned}$$

Using the approximation from Eq. 12, we then can calculate the $\varvec{u}^{-(i,j)}$ vector by setting the loading coefficients $\varvec{w}_i$ and $\varvec{w}_j$ of the pair of taxa (*i*, *j*) to zero:

13 $$\begin{aligned} \left[ \begin{array}{c} u_{1}^{-(i,j)} \\ u_{2}^{-(i,j)} \\ \vdots \\ u_{n}^{-(i,j)} \end{array}\right] _{n \times 1} \approx \left[ \begin{array}{cccc} x_{11} & x_{12} & \cdots & x_{1p}\\ x_{21} & x_{22} & \cdots & x_{2p}\\ \vdots & \vdots & \ddots & \vdots \\ x_{n1} & x_{n2} & \cdots & x_{np} \end{array}\right] _{n \times p} \times p \left[ \begin{array}{cccccccc} w_1&w_{2}&\cdots&w_i=0&\cdots&w_j=0&\cdots&w_p \end{array}\right] _{1 \times p}^{\intercal } \end{aligned}$$

This allows us to obtain the vectors ***u*** and ***w*** only once. We show the efficacy of this approximation in Appendix A.

### Comparisons to other network inference methods

Existing longitudinal network methods either infer networks for a single individual or derive a single network from all data [9], which can be problematic when we expect changes in taxa associations over time. LUPINE infers networks sequentially to include the information learned across time. We compared our results with two state-of-the-art single time point network inference methods, SpiecEasi [18] and SparCC [18], using simulations where the true network is known. SpiecEasi and SparCC represent two distinct branches of network analysis. Specifically, SparCC employs a correlation-based approach, while SpiecEasi uses a conditional dependence approach. The SpiecEasi method further includes two methods based on different graphical model estimators, representing different types of network analysis: (i) neighbourhood selection and (ii) sparse inverse covariance selection, which we will refer to as ‘SpiecEasi_mb’ and ‘SpiecEasi_glasso’, respectively.

### Measures for network comparisons

**Fig. 3:**
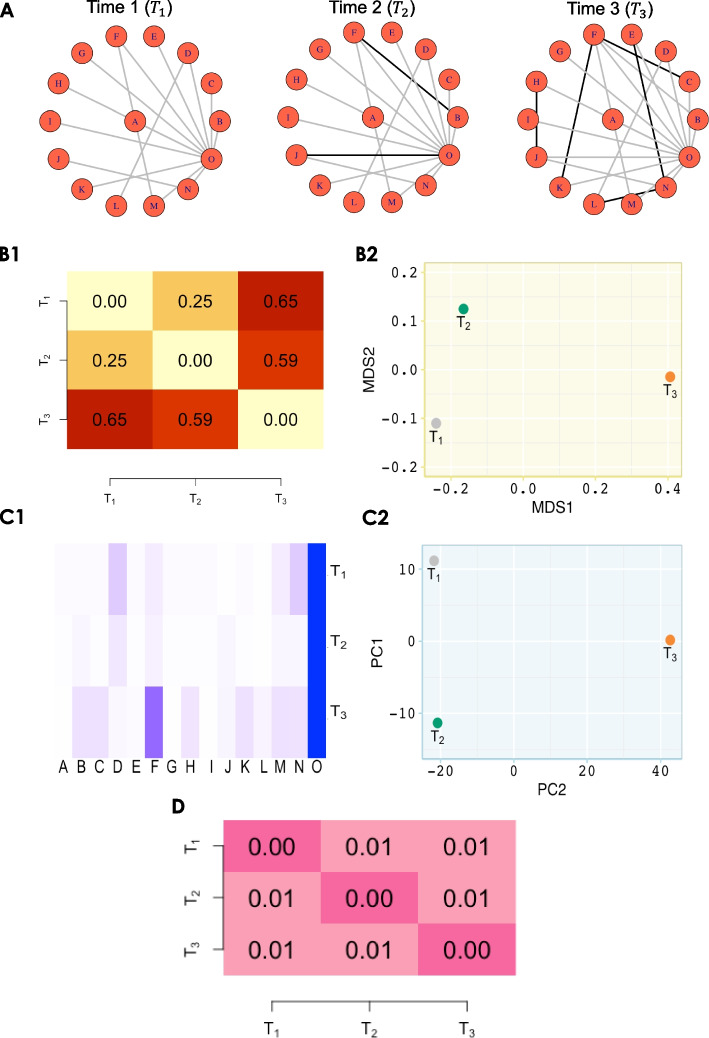
Toy example for network comparison. **A** Consider three networks across three time points, where nodes represent taxa and edges represent significant association between taxa. Black edges denote new connections compared to the preceding time point. **B** Network dissimilarity through graph diffusion distance (GDD). **B1** Pairwise GDD distances reveal that the network at $T_3$ is dissimilar to the networks at $T_1$ and $T_2$. **B2** Transformation of pairwise GDD into a lower-dimensional space using classical multidimensional scaling (MDS) represents the separation of time 3 network from the other two networks. **C** Network discrimination through influential nodes (i.e. taxa). Node influence is computed by integrating local, semi-local, and global measures. **C1** Heatmap showing the node influence (more influential = darker blue shades). For example, node ‘O’ is influential in all time points, but node ‘F’ is more influential at $T_3$ rather than $T_1$ and $T_2$. **C2** Principal component analysis (PCA) plot to represent the networks based on their respective node influence. Similar to what we observed with the GDD distance, the largest source of variation is explained between the network at $T_3$ and the networks at earlier time points. **D** A heatmap of *p*-values from the correlation tests between each pair of networks indicates that all three networks are correlated

We used two distinct measures to identify similarities and dissimilarities among the inferred networks: a network distance measure for quantitatively evaluating the network topology (see Fig. 3B1–B2) and a node-wise measure for assessing the influential nodes in the network (see Fig. 3C1–C2). Additionally, we measured the differences between network pairs using hypothesis testing (Fig. 3D).

#### Pairwise distance between networks

We use graph diffusion distance (GDD) [19] to measure pairwise differences in network typologies. GDD assesses the average similarity between two networks by exploring the information flow and connectivity within their structures, based on the heat diffusion process on graphs. The GDD score is calculated by searching for a diffusion time that maximises the Frobenius norm of the difference between the diffusion kernels. By calculating the GDD for all pairs, we obtain a pairwise distance matrix representing the network distances between pairs of graphs. We then transform these pairwise distances into a lower-dimensional space using classical multidimensional scaling (MDS) [20]. MDS maintains pairwise dissimilarities between objects. As a result, in the MDS plot, points that represent each network are deemed similar if they are positioned closely and dissimilar if they are spaced further apart. This visual technique allows us to compare multiple networks at once (Fig. 3B2).

#### Node influence

To assess influential nodes in the network, we use the integrated value of influence (IVI) algorithm that integrates local (degree centrality and ClusterRank), semi-local (neighbourhood connectivity and local H-index), and global (betweenness centrality and collective influence) measures [21]. IVI combines these measures to capture both local prominence as well as the broader impact of the nodes. After calculating the IVI values for all nodes, we perform a PCA on the resulting matrix, where the rows represent different networks, and the columns represent different nodes (here taxa). While MDS aims to preserve pairwise distances or dissimilarities as much as possible, PCA prioritise explaining the maximum variation in the data and highlighting strong patterns (Fig. 3C2).

#### Correlation test between two networks

To evaluate correlations between two network adjacency matrices, we first calculate pairwise Hamming distances for each matrix [22]. This step allows us to compare differences in connections between nodes. Next, we apply the Mantel test [23], a permutation-based method, to assess whether the correlation between these distance matrices is statistically significant. By comparing the observed correlation to a distribution generated from random permutations, the Mantel test evaluates its significance, providing a robust measure of the relationship between the two matrices.

### Simulation and case studies

In this section, we outline our simulation strategy and detail the four case studies analysed with LUPINE.

#### Simulation study

**Fig. 4:**
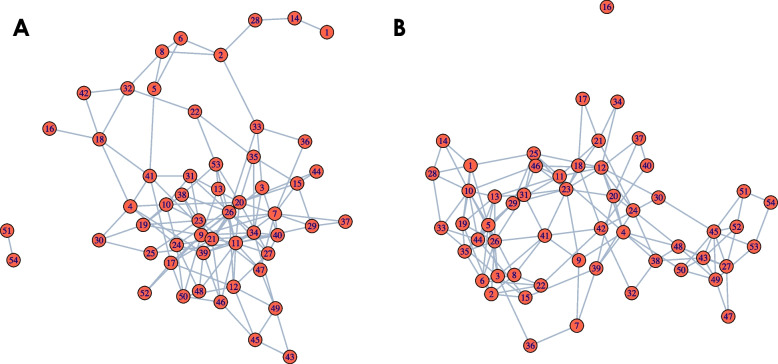
Networks used in simulation. Inferred network from SpiecEasi for **A** normal diet and **B** HFHS diet among *Bacteroidales*. These networks form the basis of our simulation for the two time periods (i.e. from day 1 to 5 and from day 6 to 10)

In our simulation, we used two realistic networks based on our high-fat high-sugar (HFHS) case study described in the Case studies section. The two networks were inferred using the SpiecEasi method [5] where concatenated the data across days for each diet. Due to computational constraints, we restricted our simulation to the sub-network of the *Bacteroidales* order, resulting in 54 nodes. The networks used across the two time periods are represented in Fig. 4. We simulated data using the network in Fig. 4A until day 5, then transitioned to the network in Fig. 4B from day 6 to day 10 to reflect a network change. We used a multivariate Poisson distribution to simulate the count data. For each simulation, a new correlation matrix was generated from the two networks. To explore the impact of sample size, we varied the sample size from 23 to 50, and then to 120. For each sample size, 50 data sets were simulated, each with 54 taxa and 10 time points. More details on the simulation strategy are provided in the Appendix B.

Using simulated data, we also conducted a sensitivity analysis in Appendix D to evaluate the robustness of LUPINE and LUPINE_single under various conditions. The analysis was designed to determine whether the model’s performance remains consistent when different parameters or assumptions are altered.

#### Case studies

We analysed three 16S rRNA amplicon datasets and one metagenomic dataset, the experimental designs of which are outlined in Table 1. For the 16S datasets which were available in raw counts, we grouped data by ‘group’ and ‘time point’ and calculated the relative abundance of each taxa within these specific groups and time points. Any taxa with a relative abundance less than 0.1% across all groups and all time points were excluded. This filtering process, while significantly reducing the number of taxa, aligns with the goal of conducting a more focused analysis [24]. However, we only calculated the partial correlations for taxa that had a mean relative abundance exceeding 0.1% at a given time point and group. This approach helps us avoid identifying connections between taxa with low abundance at certain time points in specific groups. As a result, there were no edges in the network from the taxa with low abundance at that time point and group. When we fit a longitudinal model on a given time point, we only consider the taxa filtered on that specific time point and group, and across the past time points.

**Table 1:** Summary of experimental frameworks: key parameters for each case study

Name	Sample size	Number of taxa	Time points	Collection	Interventions
High-fat high-sugar (HFHS)	HFHS = 23	212	4	Days (0, 1, 4, 7)	NA
	Normal = 24				
Vancomycin-resistant Enterococcus faecium (VREfm) colonisation	9	126	11	Days (0–2, 5–9, 12–14)	Ceftriaxone antibiotic treatment (days 6–7)
					VREfm colonisation (day 9)
Diet study	Plant diet = 10	311	15	Days (−4, −3, −2, −1, 0, 1, 2, 3, 4, 5, 6, 7, 8, 9, 10)	Diet intervention (days 0–4)
	Animal diet = 10				
Pre-diabetic study	MDE diet ^a^ = 96	91	2	Months (0,6)	Diet intervention (months 0–6)
	PPT diet ^b^ = 93				

Number of taxa are indicated after the filtering step described in the ‘Case studies’ section

^a^Mediterranean diet, ^b^Personalized postprandial glucose-targeting diet

#### Case study 1: HFHS (a four time point case-control mouse study)

We investigated the effect of a high-fat high-sugar (HFHS) diet on the mouse microbiome, as outlined in [25]. To assess the diet effect on the gut microbiome, 47 C57/B6 female black mice were fed with either an HFHS or a normal diet. Fecal samples were collected at days 0, 1, 4, and 7.

The raw data contained 1172 taxa across all four time points. We removed one outlier mouse from the normal diet group with a very large library size. After filtering, we modelled 102,107,105, and 91 taxa respectively for the normal diet for each day and 99, 147, 85, and 92 taxa for the HFHS diet.

#### Case study 2: VREfm (an eleven time point mouse study with two interventions)

Mu et al. [26] investigated the functional roles of the gut microbiome during vancomycin-resistant Enterococcus faecium (VREfm) colonisation. To assess the bacterial community composition in the murine gut, 9 C57BL/6 mice (co-housed wild-type males) were monitored. Fecal samples were collected over 14 days from the same three mice. Over the course of 14 days, the mice underwent two interventions: (1) administration of ceftriaxone treatment at a concentration of 0.5 g/liter in the drinking water over 2 days on days 6 and 7 and (2) colonisation with a dosage of $(1 \times 106)$ VREfm ST796 on day 9. The days before the antibiotic administration were considered as the naive phase of the experiment. The 2 days when the antibiotic was given were considered the antibiotic phase, and all other days after that were considered the VRE phase.

The data contained 3574 taxa across eleven time points. Day 8 was excluded from the analysis due to insufficient taxonomic counts for two mice. After filtering, we considered the following number of taxa in each phase: naive (53, 63, 58, 72), antibiotic (34, 22) and VRE (10, 15, 15, 15).

#### Case study 3: Diet (a fifteen time point case-control human study with one intervention)

David et al. [27] investigated the human gut microbiome responses to a short-term diet intervention. To evaluate the dynamics of the diet intervention, they assigned 10 participants to each diet intervention, namely the ‘plant-based’ and ‘animal-based’ diets for five consecutive days. The study participants were observed for 4 days before the diet intervention, a period referred to as the ‘baseline period’ to study their regular eating habits. After intervention, a 6-day ‘washout period’ was also observed to assess microbial recovery. Data were obtained from the MITRE repository [28]. The data contained 17,310 taxa across all time points. As taxonomic details were lacking, we used the sequence information along with dada2 reference database ‘silva_nr99_v138.1_wSpecies_train_set.fa.gz’ for taxonomic classification [29].

After filtering, we retained the following number of taxa in the plant-based diet group for each period: baseline (105, 100, 111, 103), intervention (118, 116, 116, 114, 114), and washout (121, 120, 90, 96, 115, 109); and in the animal-based diet group: baseline (120, 109, 128, 115), intervention (113, 113, 108, 105, 110), and washout (112, 104, 106, 105, 107, 123). Across the duration of the experiment, only 8–15 samples were obtained from each participant because of unsuccessful sampling at certain time points. Thus, we imputed the missing values using a cubic splines on the centred log ratio (clr) transformed data. This was motivated by the linear interpolation and cubic splines interpolation adopted in [24, 30]. Since, clr transformation accounts for library size differences, we used Eq. 3 instead of Eq. 4 in our modelling.

#### Case study 4: Pre-diabetic (large human case control study)

Shoer et al. [31] investigated the impact of a personalized postprandial glucose-targeting diet (PPT) and Mediterranean diet (MED) on gut microbiome on 200 pre-diabetic individuals. MED is the standard of care for pre-diabetes. Participants provided fecal samples before and after a 6-month diet intervention.

The data contained 378 fecal gut samples with 605 species-level genome bins (SGBs) given as unique relative abundance (URA, [32]). After removing *Archaea* and unknown kingdoms, we were left with 505 features, but with many missing values. We only kept features with less than 25% missing values across all samples, resulting in 91 SGBs.

## Results

We first evaluated our two approaches against two widely recognised network inference methods (described in the ‘Comparisons to other network inference methods’ section) for cross-sectional microbiome data. Using simulated data, our objective was to demonstrate the effectiveness and computational efficiency of our proposed approaches compared to commonly used single time point methods. We then highlighted the advantages of LUPINE over its single time point counterpart LUPINE_single in the HFHS study.

After this benchmark step, we applied LUPINE in four case studies. In the longitudinal case-control mouse study, we evaluated whether the difference in diets could be detected in our network models. In the mouse study with two interventions, we evaluated the effectiveness of LUPINE in detecting abrupt changes. In the human case-control study, we assessed the ability of LUPINE to handle inherent high variability typical of human studies across fifteen time points. Finally, in the metagenomic human case-control study, we assessed LUPINE’s capacity to generate biological inferences in a large human study with two time points.

### Benchmarking analysis

#### Simulation study: LUPINE methods outperformed existing single time point methods

We first compared our proposed longitudinal method (LUPINE) and its single time point version (LUPINE_single), with two SpiecEasi models and SparCC. Note that all methods except LUPINE were designed for single time point modelling.

We assessed model performance across 50 simulated datasets, focusing on scenarios where the number of samples was smaller than the number of variables. We used two metrics: area under the receiver operating characteristic curve (AUC-ROC) and area under the precision-recall characteristic curve (AUC-PRC). True positives were defined as the edges present in the inferred network that also existed in the simulated (true) network at each time point. For LUPINE_single, LUPINE, and SparCC, we used *p*-values from the correlation test to infer edges between pairs of nodes. A higher value for $1 - p\text {-value}$ indicated a greater likelihood of an inferred edge, with the value reflecting the strength of the connection between a pair of taxa. For the two SpiecEasi methods, stability was used to measure the strength of the inferred connection.

The AUC-ROC captures the trade-off between sensitivity (true positive rate) and specificity (false positive rate) across different thresholds ($1 - p\text {-value}$ or stability), providing a broad overview of model performance. In contrast, the AUC-PRC focuses on precision (positive predictive value) versus recall (true positive rate), which is particularly informative for scenarios where the number of true edges is much smaller than the number of non-edges. By analysing these two metrics, we gained insights into the model’s ability to distinguish true edges from spurious edges depending on network sparsity. Higher values in both metrics signified better model performance, with AUC-ROC evaluating overall discriminatory power and AUC-PRC evaluating precision and recall characteristics.

**Fig. 5:**
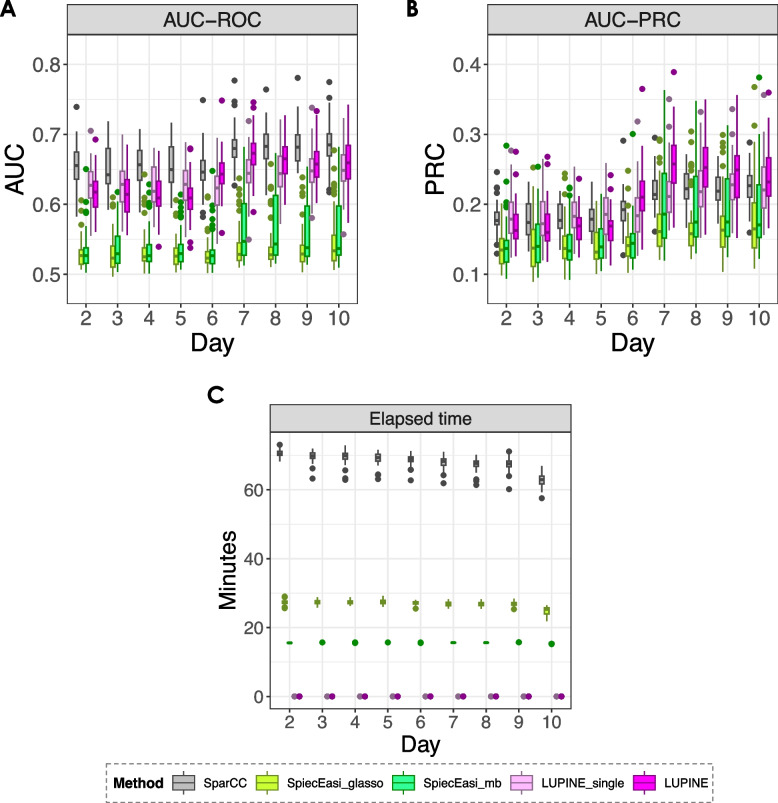
Simulation results (*n* = 23). Box plots of the ** A** area under the receiver operating characteristic (AUC-ROC), **B** precision-recall curve (AUC-ROC) over time for different methods. Although all methods performed similarly in terms of AUC-PRC values, both LUPINE models and SparCC outperformed the two SpiecEasi methods based on AUC-ROC values. **C** Elapsed time for each method, showing the superior computational performance of the two LUPINE methods

Our proposed methods and SparCC outperformed SpiecEasi with higher AUC-ROC values (Fig. 5A). However, all methods had similar AUC-PRC values (Fig. 5B). Additionally, both our approaches were computationally more efficient than SparCC (Fig. 5C). When the sample size increased, LUPINE and LUPINE_single led to superior model performance based on AUC-ROC and AUC-PRC values (Figs. 16 and 17 in Appendix C).

Next, we compared the inferred networks across time points, as explained in the ‘Measures for network comparisons’ section. For SparCC and our methods, taxa connections were determined via a *p*-value cut off (this was not required for SpiecEasi methods, as they inherently output a binary network).

**Fig. 6:**
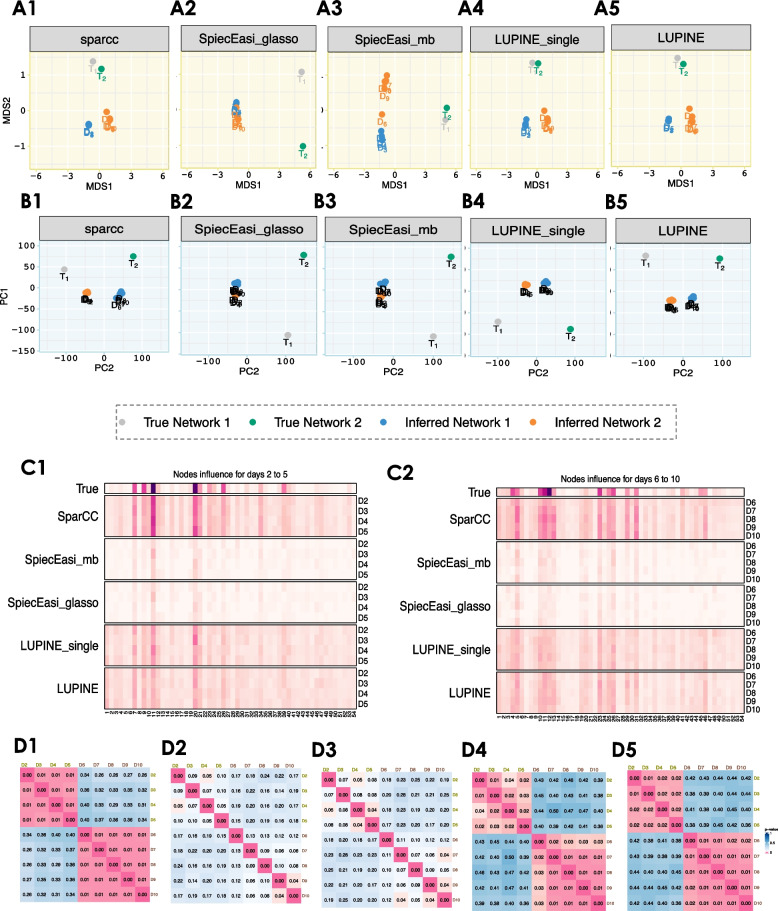
Network comparisons for simulation results *n* = 23. **A1–A5** MDS plots of the pairwise GDD for the inferred networks from the different methods. All but SpiecEasi methods distinguish networks based on the two periods (days 2–5 and 6–10) in the first coordinate, regardless of the network being true or inferred. **B1–B5** PCA plots of the IVI scores for the inferred networks for each method. The largest node influence variation occurs across days, with a clear contrast between the two periods (days 2–5 and 6–10) for both LUPINE models and SparCC. Heatmaps of mean IVI scores for each node **C1** from day 2 to 5 and **C2** from day 6 to 10. The first row displays IVI scores for true networks from Fig. 4. All methods identified the most influential nodes. **D1–D5** Heatmaps of network correlation *p*-values indicate large differences between networks from days 2 to 5 from days 6 to 10 in both LUPINE models and SparCC

To compare networks based on structure, we computed the pairwise GDDs for all inferred networks across time points, then averaged these distances for each time point across all simulations. In our simulation (described in the ‘Simulation study’ section), we generated two stable networks that were distinct, across two time periods (days 2–5 and 6–10). Thus, we expect the inferred networks to be similar within each period and dissimilar between the two periods. However, SpiecEasi displayed the largest variation between the inferred and the actual networks in the MDS plot of the pairwise GDDs (Fig. 6A2–A3). In contrast, our two proposed methods (Fig. 6A4–A5) and SparCC (Fig. 6A1) showed the largest between-period variation, regardless of whether the networks were true or inferred.

We applied the same procedure used for GDDs to conduct node influence based comparisons based on IVI values. Similar to the MDS plots, SparCC (Fig. 6B1) and LUPINE methods (Fig. 6B4–B5) explained the greatest between-period variation in the PCA plots. While all methods identified the most influential nodes (Fig. 6C1–C2), the two SpiecEasi methods showed less pronounced influential nodes due to their sparse network inference, compared to the other methods. Both LUPINE methods and SparCC successfully distinguished between the networks from days 2 to 5 and days 6 to 10 (Fig. 6D1–D5). As expected, the correlations were significant within the day 2 to 5 and day 6 to 10 intervals, but not between days within each interval.

#### HFHS study: LUPINE highlighted more robust longitudinal network patterns than LUPINE_single

We compared LUPINE and LUPINE_single on the HFHS study. This study includes a small number of time points (Fig. 8A), where mice were subjected to either a HFHS or a normal diet.

**Fig. 7:**
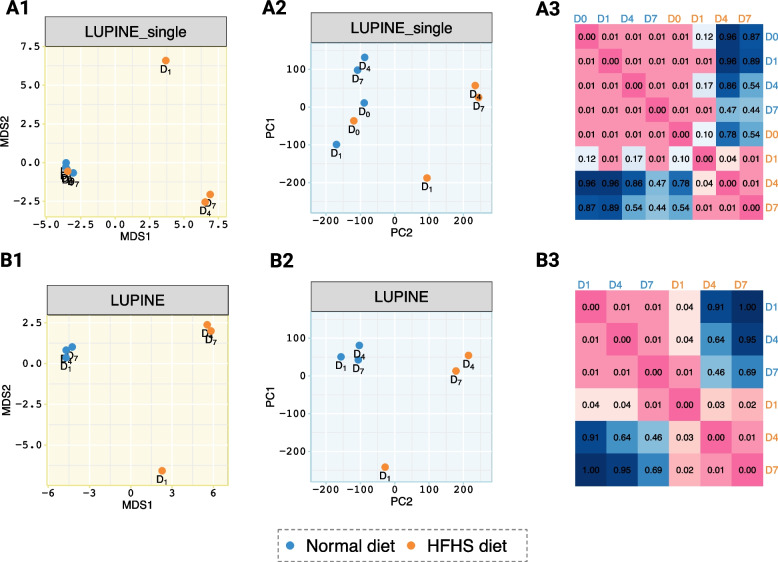
Network comparison results for LUPINE_single and LUPINE. **A1–B1** MDS plot of pairwise GDD and **A2–B2** PCA plot of the IVI scores illustrating network similarities. **A** from LUPINE_single scheme and **B** from LUPINE longitudinal scheme. Compared to LUPINE_single, LUPINE exhibits consistent grouping patterns for both types of measures. **A3–B3** Heatmaps of network correlation *p*-values show that both approaches effectively identify significant differences between networks in the normal diet group and the later-stage networks in the HFHS diet group

Both LUPINE_single and LUPINE differentiated the diet groups in the first axis of the MDS and PCA plots (Fig. 7). For LUPINE_single, microbial networks from the normal diet group and the HFHS diet at day 0 were clustered, indicating a similar network structure at the onset of the two different diets (Fig. 7A2). However, we did not observe a close cluster of microbial networks within the normal diet networks in the PCA plot of the IVI scores (Fig. 7A2), suggesting that the node influence within the normal diet group is changing over time.

In contrast, for LUPINE, the groupings in the MDS plot (Fig. 7B1) closely matched to those observed in the PCA plot (Fig. 7B2), indicating consistent network results based on both GDD and IVI scores. Thus, these results suggest that LUPINE is able to model robust longitudinal patterns. Note that the initial day 0 microbial network is absent from the longitudinal scheme as we require at least one prior time point for inference. However, we found that both approaches successfully identified significant correlations within each diet group across days, while correlations between diet groups across days were insignificant, particularly later in the diet (Fig. 7A3–B3).

In this benchmarking section, we first demonstrated the superior model performance of both LUPINE and LUPINE_single using AUC-ROC and AUC-PRC values. We then highlighted the advantages of LUPINE in comparison to our own LUPINE_single approach. We showed that LUPINE led to robust longitudinal network patterns. The following sections focus on the biological interpretation of the microbial communities identified by LUPINE in four case studies.

### HFHS study: LUPINE identified taxonomic orders that differentiate microbial networks between different diet groups in mice across four time points

**Fig. 8:**
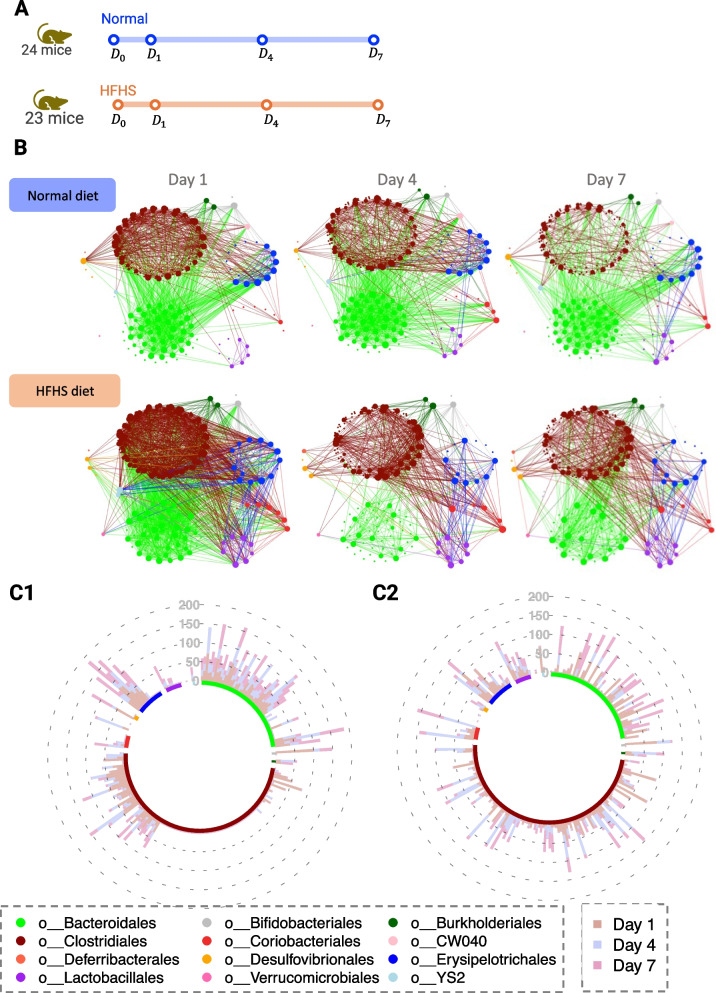
HFHS case study. **A** Graphical representation of the study timeline. **B** Inferred microbial networks over time for both normal group and HFHS diet groups. In the HFHS diet group, we observe a decrease in connections among nodes belonging to the *Bacteroidales* order over time. In the normal diet group, nodes belonging to the *Erysipelotrichales* order are more connected to those from the *Bacteroidales* order, but in the HFHS diet group, we observe that nodes belonging to the *Erysipelotrichales* order are more connected to those from the *Lactobacillales* order. **C** Circular stacked bar plot of the IVI scores for **C1** normal diet and **C2** HFHS diet groups show a reduction in IVI scores for nodes associated with the *Clostridales* order in the normal diet group compared to the HFHS diet group

We identified patterns in the microbial network plots that distinguished the two diet groups (Fig. 8B). In the normal diet group, we observed denser connections among nodes within the *Bacteroidales* order, particularly on days 4 and 7, compared to the HFHS diet group. Additionally, nodes within the *Lactobacillales* order exhibited a higher number of connections in the HFHS diet group, specifically with nodes within the *Erysipelotrichales* and *Clostridales*. Similar inferences can also be made from the IVI scores in Fig. 8C1–C2: in the normal diet group, we observed decreased IVI scores across days for nodes within the *Clostridales* order, with the majority having a zero IVI value. Compared to the normal diet group, the HFHS diet group networks had a higher node influence in *Lactobacillales* order (Fig. 8C2). While these findings do not imply causation, they are consistent with previous studies that reported decreased relative abundances of *Bacteroidales* and enrichment of *Lactobacillales* in mice that had been fed with a high-fat diet [33, 34], suggesting a potential influence of diet on these taxonomic orders. Daniel et al. [35] also found that the proportions of *Lactobacillales* and *Erysipelotrichales* were higher in mice fed with high-fat diet. *Lactobacillus* has also been extensively studied to prevent or treat type 2 diabetes mellitus [36], indicating its potential effect on high sugar diet.

To summarise, LUPINE analysis highlighted two different microbial community networks between normal and HFHS diets, with an increase in connections in the taxa nodes belonging to *Lactobacillales* in mice that were fed with a HFHS diet.

### VREfm study: LUPINE highlighted changes in the network structure across two abrupt interventions in a mouse study with 11 time points

**Fig. 9:**
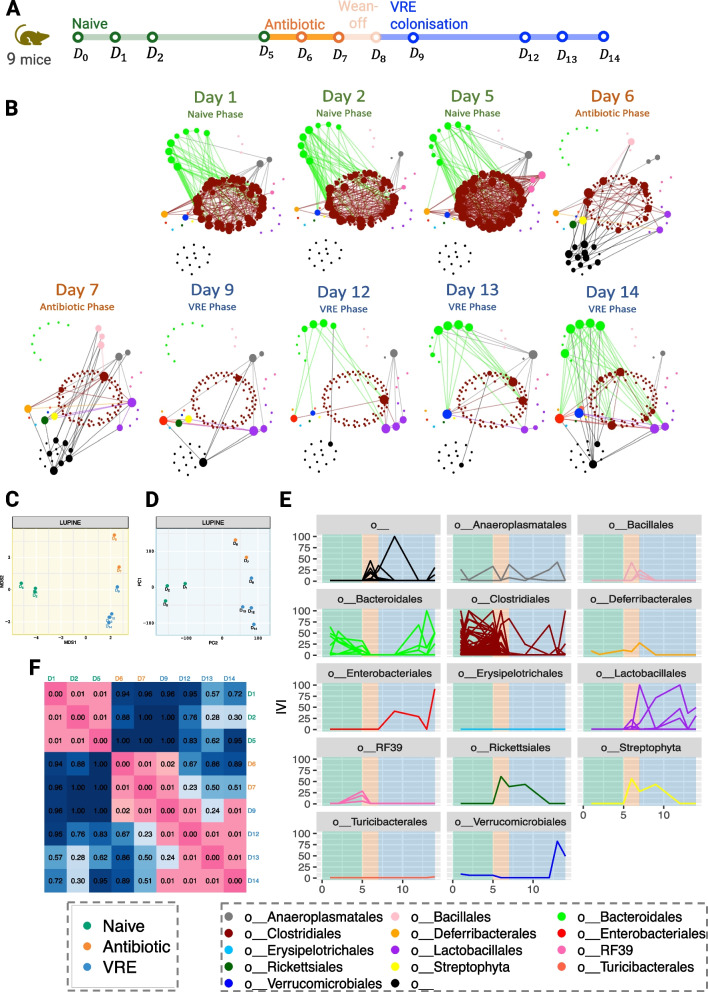
VREfm case study. **A** Graphical representation of the study timeline. **B** Inferred networks across time. After antibiotic treatment on day 6, we observe a reduction in the associations in the nodes belonging to the *Clostridales* order, which are not recovered until day 14. However, the connections among nodes of the *Bacteroidales* order appear to recover from day 12. **C** MDS plot of the pairwise GDD shows that the network structures are significantly different for each phase of the experiment. **D** PCA plot of the IVI scores exhibits a grouping pattern consistent with the MDS plot. **E** Line plots of the IVI scores for each taxa across time, grouped by their taxonomic order show a reduction of influential nodes after antibiotics treatment, indicating a less dense network structure. **F** The heatmap of Mantel *p*-values comparing network correlations indicates significant correlations within the naive phase, between the antibiotic phase and day 9, and across the last three days of the VRE phase

In the VREfm (Fig. 9A), mice underwent two interventions: antibiotic administration and VRE colonisation. Similar to the first case study, this is a more controlled experiment compared to human studies. However, in this study, the time periods are divided into three phases: naive, antibiotic, and VRE, reflecting different stages of the experiment. In contrast to the first case study, where the objective was to identify the group differences the objective here is to explore network differences between the phases.

During the antibiotic phase, the number of edges in the inferred networks decreased in *Bacteroidales* and *Clostridiales* (Fig. 9B). *Bacteroidales* are an important microbe for short-chain fatty acids. Miao et al. [37] found that *Bacteroidales* almost disappear from mouse feces during ceftriaxone treatment. During the VRE phase, the number of edges increased, but this increase was not observed across all taxonomic orders. Specifically, we observed an increased number of edges in the nodes belonging to *Bacteroidales*. In contrast, and even at the end of the experiment, the nodes belonging to *Clostridiales* had very few connections compared those detecting in the naive phase. From the antibiotic phase to day 9, two nodes belonging to taxonomic order *Streptophyta* and *Rickettsiales* exhibited higher connections. This finding is in agreement with [38], who studied the disruption of the gut microbiota after the administration of broad-spectrum antibiotics and found a strong relative increase in bacteria of the genus *Streptococcus* across all subjects. Therefore, the increased connections of *Streptophyta* and *Rickettsiales* could potentially be attributed to the impact of antibiotics on the gut microbiota. From day 12, we also observed an increased number of connections in *Verrucomicrobiales*. The genome of *Akkermansia muciniphila*, a species within this taxonomic order, has been examined for its potential antibiotic resistance-associated genes [39].

The MDS plot of the pairwise GDD revealed a clear separation between the naive phase, antibiotic phase, and VRE phase networks (Fig. 9C). Within the VRE phase, we observed a temporal progression, indicating a unique network structure on day 9, which marked the initiation of VREfm colonisation. The PCA plot of the IVI scores (Fig. 9D) showed a similar grouping structure to the MDS plot. The similarity between the two plots indicated a consensus in the network grouping, regardless of the two metrics employed (as we showed previously in the HFHS study). The naive phase networks IVI scores were well separated from the other phases, accounting for 35% of total variation of the scores. After the naive phase, we observed a decrease in the influential nodes for the majority of taxa. However, taxa belonging to the *Enterobacterales* and *Lactobacillales* orders became more influential following the antibiotic phase (Fig. 9E). In [9], we also found that these taxa belonging to families *Enterobacteriaceae* and *Enterococcaceae* showed a group difference between the naive and the VRE phases. We also noted a decrease in the IVI scores for *Clostridiales* nodes following the naive phase, a decrease that persisted until the end of the experiment. This taxonomic order was a key taxa group that has been studied for its ability to restrict gut colonisation by *Enterobacteriaceae* pathogens in mice, as shown by [40–42]. When comparing networks, we observed strong correlations within the naive phase, between the antibiotic phase and day 9, and during the later days of the VRE phase (Fig. 9F). Additionally, there was a weak correlation between the networks across phases.

**Fig. 10:**
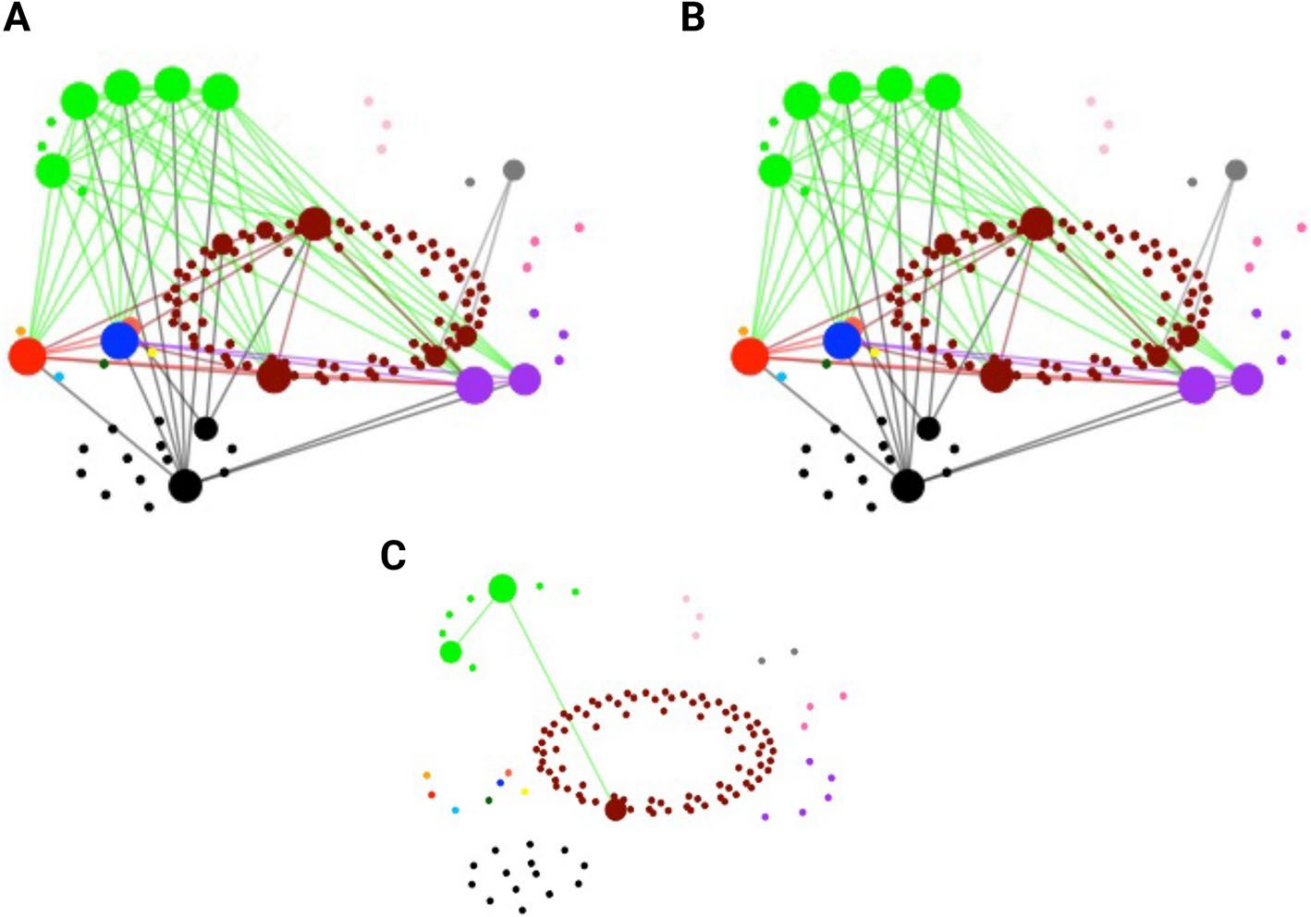
Impact of past data on the day 14 microbial network in the VREfm case study. **A** Inferred microbial network at day 14 using all past time points. **B** Inferred microbial network at day 14 using only the VRE phase time points. **C** Difference between the two networks, highlighting only two edges present in A but absent in B. The two networks are largely similar

Due to the weak correlation between the VRE phase and the other two phases, we evaluated how the amount of past information can affect the day 14 network structure (using data from day 9 onward vs. data from all past time points). The network based solely from the VRE phase data had only two fewer edges (Fig. 10B) than the network built based on all time points (Fig. 10A). The two networks differed only by two edges: one between *Bacteroidales* species and another between *Bacteroidales* and *Clostridiales* (Fig. 10C). This finding suggests that using all prior data or only VRE phase data has minimal impact on the network structure. However, users can adjust how many past time points they may wish to include in the model.

To summarise, LUPINE highlighted a reduction in the number of edges after antibiotic treatment, affecting nodes belonging to the *Clostridiales* order. Interestingly, the reduction in *Clostridiales* node connections persisted even after the antibiotic phase, suggesting that the VRE colonisation had an impact on the recovery of these connections. However, further studies are needed to assess whether these connections would be recovered without VRE intervention.

### Diet study: LUPINE detected diet-specific and diet-stable taxonomic groups in a case-control human study spanning accross 15 time points

**Fig. 11:**
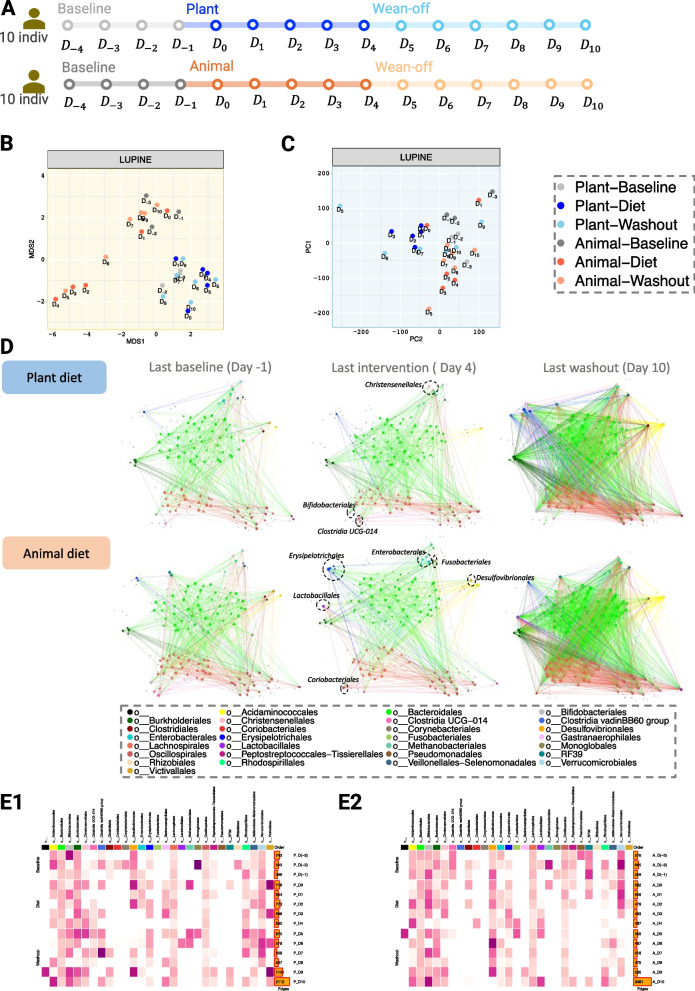
Diet case study. **A** Graphical representation of the study timeline. **B** MDS plot of pairwise GDD shows that distinct patterns emerge based on diet groups (tighter cluster for plant-based networks compared to animal-based networks). **C** PCA plot of the IVI scores show that inferred networks at baseline are similar regardless of diet, indicating similar node wise importance. **D** Inferred networks across two diet groups and three time points (last day of baseline, intervention and washout phase). On day 4, the plant-based network shows increased connections in nodes associated with *Christensenellales*, *Christensenellales*, and *Clostridia UCG 014* compared to the animal-based network, which exhibits increased connections in nodes related to *Erysipelotrichales*, *Lactobacillales*, *Coriobacteriales*, *Enterobacterales*, *Fusobacteriales*, and *Desulfovibrionales*. **E** Heatmap of the average IVI score for each taxonomic order for **E1** plant based and **E2** animal based diet groups, with darker pink representing high average IVI value. Nodes from *Bacteroidetes*, *Lachnospirales*, and *Oscilospirales* consistently exhibit a non-zero IVI score, indicating their stable influence, unaffected by diet or daily variations

The diet case-control study differs from the previous ones as it involves humans and numerous time points (Fig. 11A) and a small number of participants. This results in expected higher variability than mouse studies.

The plant-based diet group differed from the animal-based diet group, with the former showing a tighter network grouping, indicating more significant changes in the animal diet intervention (Fig. 11B). In the animal diet group, two clusters were observed. The first included networks from days 2 to 4, and the second from days − 3 to 1 and 7 to 10, with the network on day 6 distinct from both clusters. This suggests that the network structure returned to its pre-intervention form two days into the washout period. A high variability was also noted across the animal diet group networks in the PCA plot (Fig. 11C). However, the PCA plot revealed a less distinct separation between the two network groupings within the animal diet group compared to the MDS plot. This indicates a larger difference in the inferred networks, due to information flow or structure, compared to the IVI score variation. Furthermore, we noticed a close alignment in the baseline inferred networks across both groups. This implies a similar degree of node influence during the baseline phase for both groups.

We examined in more detail the six inferred networks from the two diet groups at three different days, each reflecting the final day of each phase: baseline, intervention, and washout (Fig. 11D). We highlighted few taxa nodes in the networks at day 4 that differed between the two diet groups. On day 4, we observed the nodes belonging to *Clostridia UCG 014* had a comparatively higher degree in the plant based diet group than in the animal based diet group. *Clostridia UCG 014* is a bacterial taxon belonging to the class *Clostridia* in the phylum *Firmicutes*. While [43] found that *Clostridia UCG 014* was reduced in the high-fat diet, [44] associated the decrease in *Clostridia UCG 014* with obesity. Additionally, [45] found a positive correlation between *Clostridia UCG 014* and blood glucose levels, and [46] inferred *Clostridia UCG 014* as one of the taxa potentially linked to cholesterol regulation. [47] found *Clostridia UCG 014* to be positively correlated with propionate in mice fecal samples. Propionate is a short-chain fatty acid that can be produced by gut bacteria through fermentation of dietary fiber. Additionally, *Clostridia UCG 014* was also found to be co-exist with other fiber responsive bacteria such as *Ruminococcus* [48]. Nodes belonging to *Lactobacillales* had comparatively higher degree in the animal based diet group compared to the plant based diet group. The original study also found that the abundances of *Lactococcus lactis* and *Pediococcus acidilactici*, which belong to the taxonomy order *Lactobacillales*, significantly increased in the animal-based diet. This is in agreement with the review from [49] reporting an increased level of *Lactobacillus*, a member of the *Lactobacillales* order, resulting from the consumption of white meat protein (such as chicken and fish) and dairy products (like milk, cheese, yogurt, and kefir). By comparing these taxa with the average IVI scores across the intervention period, we observed some agreement between a high average IVI score (Fig. 11E1–E2) and a high number of connections in the networks in Fig. 11D. For instance, *Clostridia UCG 014* had a non zero IVI average in the plant based diet group from day 2, whereas the IVI score in the animal based diet group was zero specifically during the intervention period (day 0 to day 4). We also observed that the nodes belonging to taxa order *Lactobacillales* had a non zero IVI average in the animal based diet group from day 2, whereas the IVI score in the plant based diet group was zero specifically during days 1 to 5. In the heatmaps, the bar plots revealed a high number of edges on the last day (i.e. day 10) compared to all other days in both groups. No clear reasoning can be given for this observation as there was no change at this particular time point. However, this network did not stand out in our network comparison, indicating that even though the number of connections increased, the network structure and the influential nodes on day 10 were similar to those on day 9. A densely connected network at the last time point was also visible in the network plots in Fig. 11D. However, unlike the previous two case studies, we did not find diet specific correlations occurring in this particular case study (Fig. 18 in Appendix C).

To summarise, LUPINE applied to a case-control human study with intervention identified several taxonomic associations that were specific to plant based diet group or animal based diet group. We also identified few taxonomic orders such as *Bacteroidetes*, *Lachnospirales*, and *Oscilospirales* that were influential regardless of time or diet intervention. This suggests that these taxa may exhibit stability in the face of dietary changes. In fact, *Oscillospira* is currently being explored as a next-generation probiotic due to its potential health benefits [50].

### Pre-diabetic study: LUPINE identified key taxonomic groups studied as potential therapeutic targets for diabetic patients in a large human metagenomic study conducted at two time points.

**Fig. 12:**
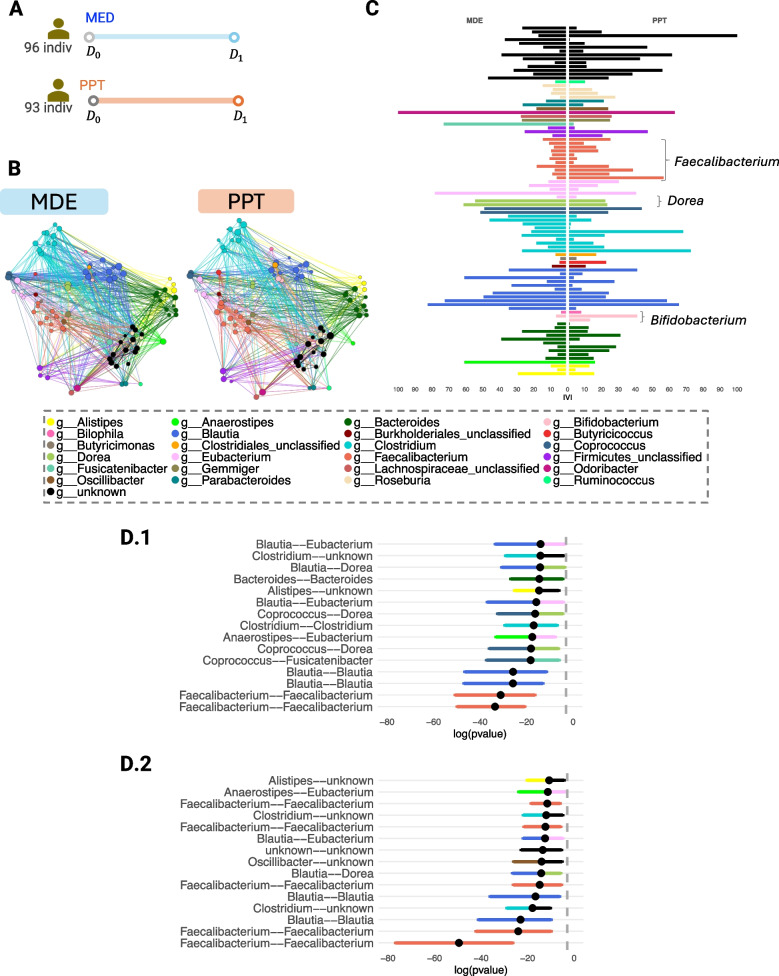
Pre-diabetic case study. **A** Graphical representation of the study timeline. **B** Inferred networks post-intervention for MDE and PPT diets show similar network structures across both diets. **C** Bar plot of IVI scores for each SGB highlights subtle differences in some genus groups. Notably, nodes belonging to *Bifidobacterium* and *Faecalibacterium* exhibit higher IVI scores in the PPT diet compared to the MDE diet. Confidence interval plots for log transformed *p*-values from correlation tests: **D1** MDE diet **D2** PPT diet, show a higher number of connections between *Faecalibacterium* pairs in the PPT diet compared to the MDE diet. The dashed grey vertical line represents a *p*-value of 0.05

**Fig. 16:**
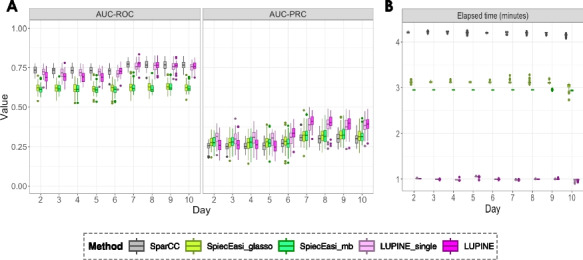
**A** Box plots of the area under the receiver operating characteristic (AUC-ROC), precision-recall curve (AUC-PRC) and **B** elapsed time values across different network inference methods for sample size 50. Each boxplot represents a distinct method, distinguished by different colours. Similar to sample size 23, two LUPINE methods and SparCC outperformed the two SpiecEasi methods based on AUC-ROC values. However, two LUPINE methods outperformed SparCC, specifically in later days. Similar to sample size 23, elapsed time was superior in LUPINE methods

**Fig. 17:**
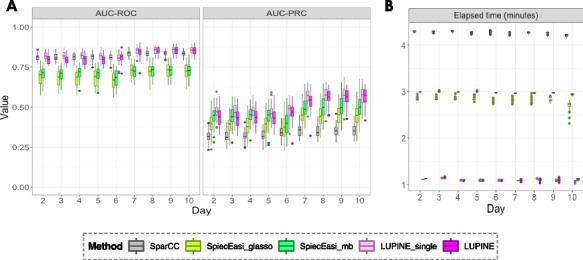
**A** Box plots of the area under the receiver operating characteristic (AUC-ROC), precision-recall curve (AUC-PRC) and **B** elapsed time values across different network inference methods for sample size 120. Each boxplot represents a distinct method, distinguished by different colours. Similar to sample size 23 and 50, two LUPINE methods and SparCC outperformed the two SpiecEasi methods based on AUC-ROC values. However, two LUPINE methods outperformed all other methods based on AUC-PRC values. Elapsed time was again superior in LUPINE methods

**Fig. 18:**
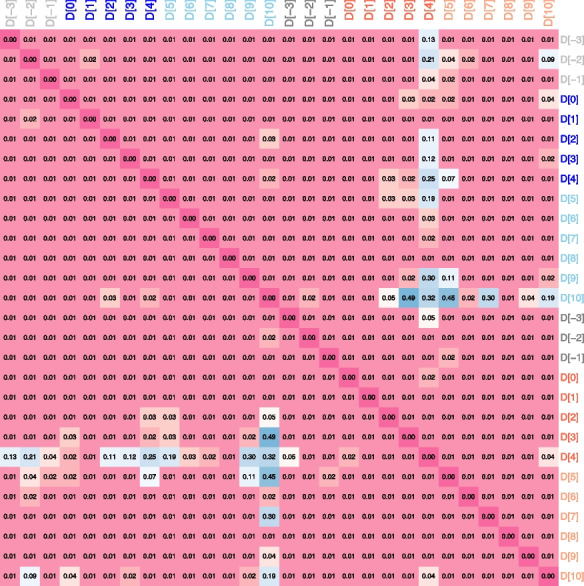
Heatmap of Mantel *p*-values for diet case study. In both animal and plant diets, there is a significant correlation across networks, indicating the presence of a core network shared between the two diets

The pre-diabetic case study includes metagenomics data from a larger number of individuals in each group, across two time points (Fig. 12A), with pre-diabetic individuals following either a MED diet or a PPT diet.

Compared to other case studies, the networks inferred through LUPINE displayed a similar network structure in the pre-diabetic case study (Fig. 12B). This was further confirmed by a low *p*-value in the Mantel test, indicating a significant correlation between the network adjacency matrices. However, we identified differences in the two networks based on IVI scores (Fig. 12C). Specifically, several genera varied between the two diet groups. For example, nodes belonging to the genus *Faecalibacterium* had higher IVI scores in the PPT group compared to the MED group. Previous research by [51] showed that butyrate-producing bacteria, such as *Faecalibacterium prausnitzii*, were reduced in individuals with pre-diabetes compared to those with normal glucose tolerance. Several other studies have also reported a decline in butyrate-producing bacteria in pre-diabetic or diabetic groups [52, 53]. Butyrate has been shown to downregulate inflammation and increases mucosal barrier integrity [54]. Palacios et al. [55] suggested that increased butyrate levels could enhance glucose management, while [56] demonstrated that *Faecalibacterium prausnitzii* lowered fasting blood glucose, improved glucose tolerance, and reduced $\text {HbA}_{\text {1c}}$ levels in pre-diabetic and diabetic mice. Thus, a higher number of connections for nodes belonging to *Faecalibacterium* may indicate positive effects of the PPT diet on pre-diabetic patients.

Similarly, nodes belonging to *Bifidobacterium* also had higher IVI scores in the PPT group than in the MED group. Chang et al. [57] found lower levels of *Bifidobacterium* in pre-diabetic patients compared to healthy individuals. *Bifidobacterium* was observed as a bacterium that is indirectly capable of promoting GLP-1 (glucagon-like peptide-1) production [58]. GLP-1 receptor agonists are used as a class of medication to treat type 2 diabetes [59]. Therefore, higher IVI scores for *Bifidobacterium* nodes may further suggest beneficial effects of the PPT diet on pre-diabetic patients. In contrast, nodes from *Dorea* had lower IVI scores in the PPT group than in the MED group. Several studies have reported higher abundance of *Dorea* in pre-diabetic individuals [60–62]. Additionally, [62] identified *Dorea* as one of the top 10 bacteria differentiating healthy individuals from diabetic patients (type 2 or pre-diabetic). Positive correlations were also found between *Dorea* abundance and fasting plasma glucose, C-peptide, BMI, and waist circumference [60]. Hence, lower IVI scores for *Dorea* nodes may indicate a healthier profile.

We also observed differences between the two diet groups based on these bootstrap confidence intervals for the *p*-values from correlation tests between pairs of taxa (Fig. 12D1-D2). In the PPT diet group, most of the 15 lowest *p*-values indicating significant associations were between nodes belonging to *Faecalibacterium*. *Faecalibacterium prausnitzii*, a species from this genus, has been identified as a potential target for diabetes prevention or treatment strategies, such as prebiotics or probiotics, due to its role in producing butyrate, which enhances insulin homeostasis [63]. Therefore, increased connections involving *Faecalibacterium* could reflect the positive effects of the PPT diet. In the MDE diet group, nodes belonging to *Dorea* were associated with *Coprococcus* nodes twice and with a *Blautia* node once. In the PPT diet group, *Dorea* was only associated with a *Blautia* node in the top 15 associations. Takeuchi et al. [64] showed that organisms from the genera *Blautia*, *Dorea*, and *Coprococcus*, as listed in the Kyoto Encyclopedia of Genes and Genomes, exhibited the three highest positive correlations with fecal carbohydrates.

To summarise, LUPINE applied to a large case-control metagenomic human study indicated that both MDE and PPT resulted in similar networks. However, downstream analysis highlighted several taxonomic genera that were more prominent in the PPT diet, including *Faecalibacterium*. *Faecalibacterium prausnitzii*, a species within this genus, has been suggested as a therapeutic option for type 2 diabetes due to its potential to enhance insulin sensitivity, improve lipid metabolism, and reduce inflammation [65].

## Discussion

We developed LUPINE to detect the stability of taxa associations within a microbial community over time and the responses of these microbial communities to external disturbances such as dietary changes and medication. To the best of our knowledge, LUPINE is the first sequential microbial network inference approach for a longitudinal setting.

In LUPINE, we combined the concepts of low-dimensional approximation with partial correlation to infer networks across time points. We then used GDD and IVI metrics to identify any abrupt network changes across time, groups, and key taxa nodes in each of the networks. Additionally, we tested the significant correlations between network adjacency matrices using the Mantel test. Note that a requirement for LUPINE is that samples match across time points, as LUPINE assumes correlation structure across time and individuals. In the case where different individuals are sampled across time, a better approach for network inference is our variant LUPINE_single applied to each time point.

In our simulation study, we demonstrated that LUPINE and LUPINE_single improved model performance and were computationally more efficient compared to existing network modelling approaches SparCC and SpiecEasi that were designed for single time point analyses. We also demonstrated the applicability of LUPINE in case studies with different study designs. The two controlled mouse studies were either case-control or intervention studies, while the third and fourth studies were more complex human study incorporating elements of both a case-control and an intervention design in either long or short time courses. In all case studies, our LUPINE analyses extracted meaningful biological insights, including clustering patterns in the inferred networks that were coherent within each study design.

While developing LUPINE, we had to consider several aspects related to the characteristics of microbiome data. First, taxa filtering is an important data processing step in this kind of analysis. We only retained taxa with a mean relative abundance exceeding 0.1% for any group at any time point to obtain a consistent set of taxa being examined across various time points and groups. Further, to avoid identifying connections between taxa with low abundance at certain time points in specific groups, we then only examined taxa with a mean relative abundance greater than 0.1% within each group and time point in the inferred networks. Second, most experimental microbiome studies are typically low sample size, which are likely to result in false positive associations. We used a correlation test that is appropriate for small sample size when calculating test statistics. We chose to identify significant associations based on *p*-values with an arbitrary cutoff of 0.05, rather than a resampling based model selection approach (used in SpiecEasi, [5]) or a bootstrap-based *p*-value calculation approach (used in SparCC, [8]) that are computationally expensive. Adjustments for false discovery on *p*-values could also be considered to obtain sparser networks. However, our experience has shown that these adjustments may lead to empty networks in some situations.

We have identified several potential extensions of LUPINE that could enrich longitudinal analysis of microbiome data. Firstly, when considering studies with several groups (e.g. HFHS and Diet studies), LUPINE must be applied to each group separately if we assume that the true networks within these groups differ from one another. A potential extension of LUPINE could include all groups together to infer a common network across all groups. Secondly, LUPINE main focus is to identify associations between taxa at specific time points, while taking into account information from previous time points. Therefore, we do not model time gaps between points. Future developments could include approaches that weight closer time points more heavily than distant ones. While we have only analysed one metagenomic study, which focused on taxonomy profiles based on species abundance, a future direction could be to extend this approach to include functional profiles derived from gene abundance information. An interesting extension of LUPINE to fully harness these complex data would be to establish connections between these two layers, akin to multilayer networks at each time point. This advancement could significantly broaden our understanding of microbiome dynamics, opening up new possibilities for research and discovery.

## Figures and Tables

**Fig. 13:**
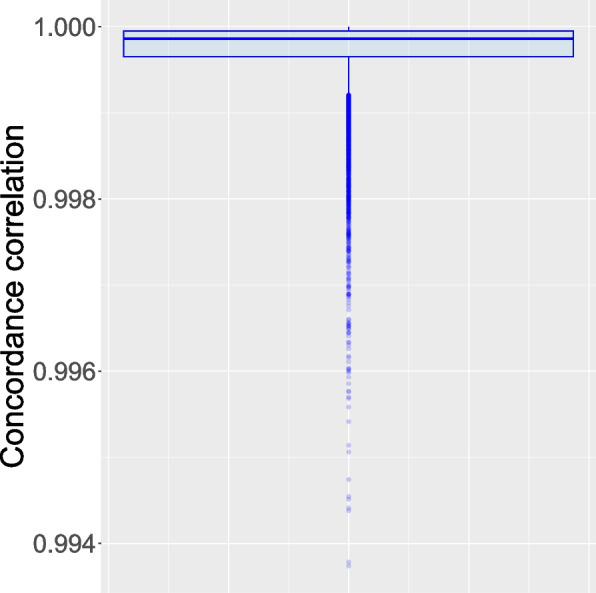
Concordance correlation values for the normal diet data on day 0 from the HFHS case study. The concordance correlation value is used to assess the agreement between the original and the approximated principal component for all excluded taxa pairs. All the correlation values are above 0.99 indicating a high level of agreement between the two approaches

**Fig. 14:**
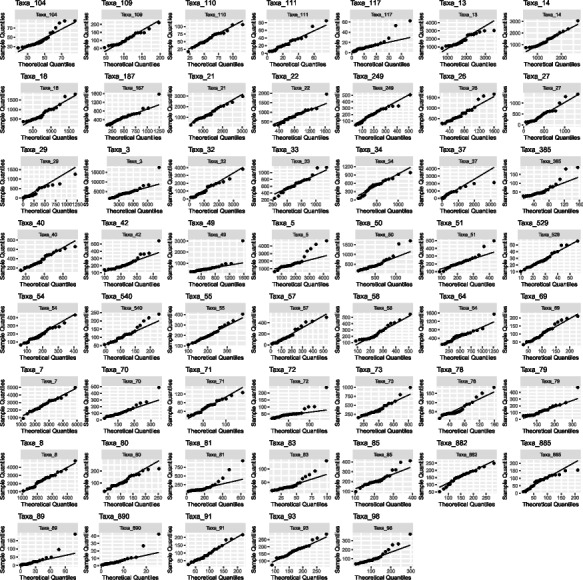
Goodness fit of the mean taxon levels estimated from gamma distributions in the normal diet group. For each taxon, qq plots are generated from theoretical quantiles and sample quantiles. Overall the fit of gamma distribution is satisfactory except for a few that deviate more from the theoretical quantiles (i.e. $Taxa_5$)

**Fig. 15:**
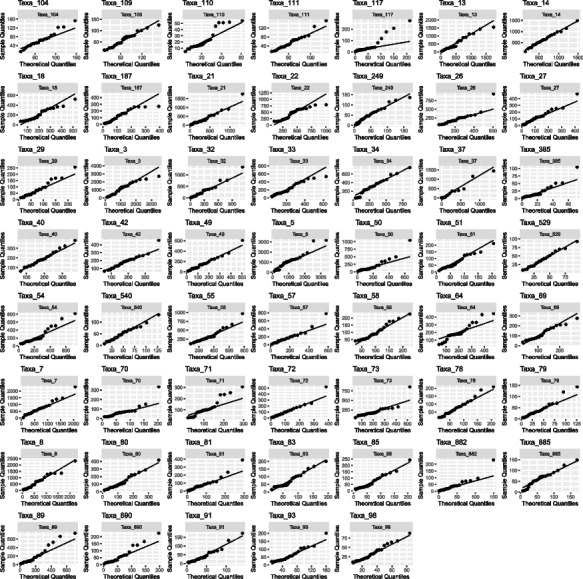
Goodness fit of the mean taxon levels estimated from gamma distributions in the HFHS diet group. For each taxon, qq plots are generated from theoretical quantiles and sample quantiles. Overall the fit of gamma distribution is satisfactory except for a few that deviate more from the theoretical quantiles (i.e. $Taxa_{117}$)

**Fig. 19:**
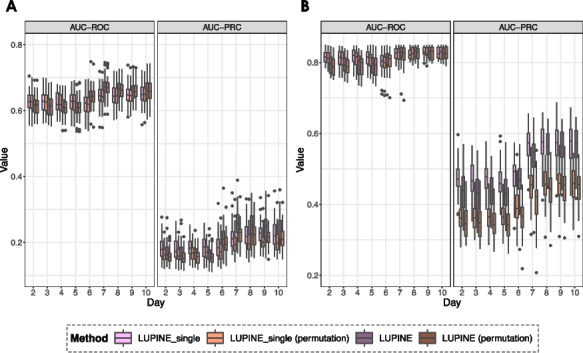
Simulated data. Box plots of the area under the receiver operating characteristic (AUC-ROC), precision-recall curve (AUC-PRC) for **A** sample size = 23 **B** sample size = 120. Each boxplot represents LUPINE_single and LUPINE evaluated with a correlation test or a permutation test, distinguished by different colours. When sample size is 23, both evaluation methods perform similarly. However, correlation based association inference outperform the permutation based inference when sample size is large

**Fig. 20:**
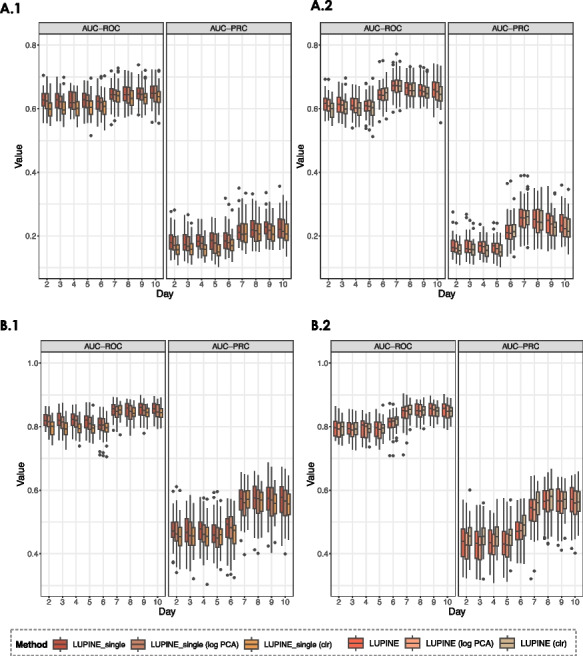
Simulated data. Box plots of the area under the receiver operating characteristic (AUC-ROC), precision-recall curve (AUC-PRC) for **A.1** sample size = 23 for different versions of LUPINE_single; **A.2** sample size = 23 for different versions of LUPINE; **B.1** sample size = 120 for different versions of LUPINE_single; **B.2** sample size = 120 for different versions of LUPINE. Each boxplot represents LUPINE_single and LUPINE evaluated with different inputs (e.g. clr tranformed vs raw counts), distinguished by different colours. For both sample sizes, all three approaches produce similar results

**Fig. 21:**
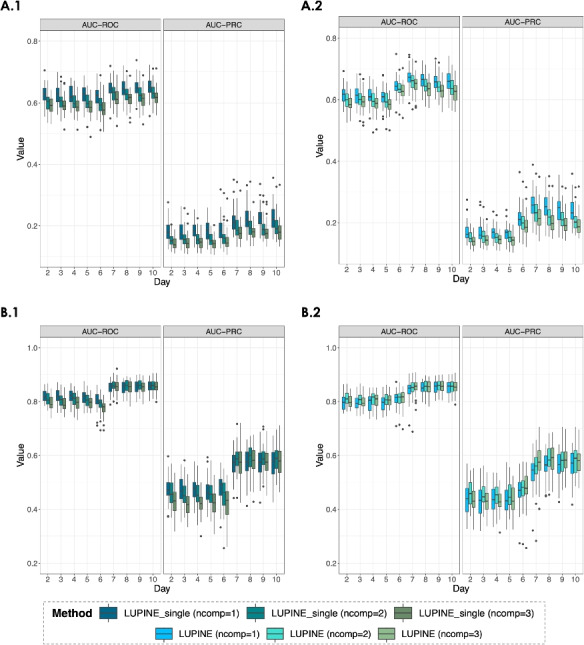
Simulated data. Box plots of the area under the receiver operating characteristic (AUC-ROC), precision-recall curve (AUC-PRC) for **A.1** sample size = 23 for LUPINE_single; **A.2** sample size = 23 for LUPINE; **B.1** sample size = 120 for LUPINE_single; **B.2** sample size = 120 for LUPINE with one to three components used in deflation. Each box plot compares the performance of LUPINE_single and LUPINE with different numbers of components used in deflation. Colours differentiate between the number of components. Results are similar for both sample sizes when using one, two, or three components

**Fig. 22:**
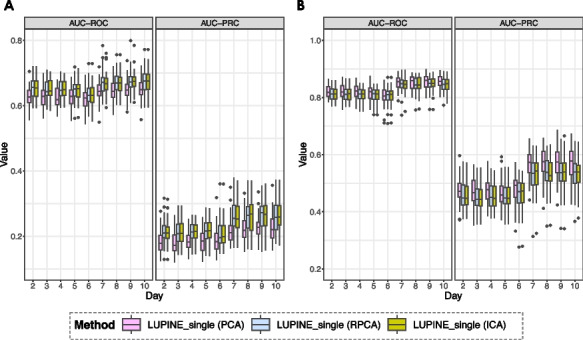
Simulated data. Box plots of the area under the receiver operating characteristic (AUC-ROC), precision-recall curve (AUC-PRC) for **A** sample size = 23 **B** sample size = 120. Each boxplot represents LUPINE_single evaluated with a PCA, RPCA, and IPCA, each distinguished by different colours. All three dimension reduction approaches produce similar results

**Fig. 23:**
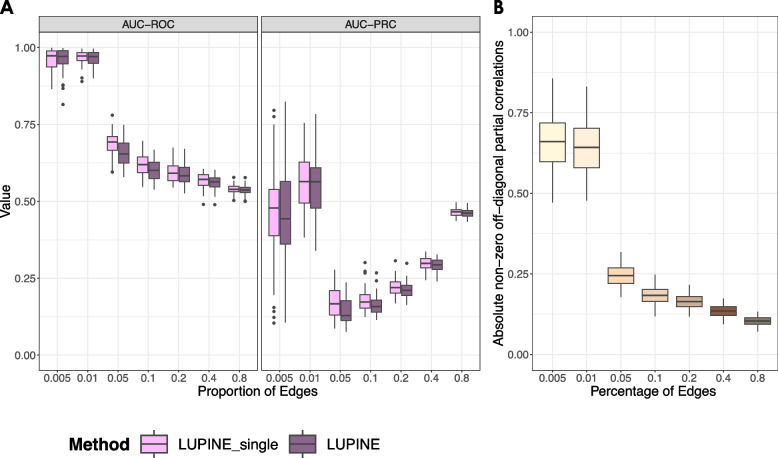
Simulated data. Box plots of the **A** area under the receiver operating characteristic (AUC-ROC), precision-recall curve (AUC-PRC) for different percentages of edges with a sample size = 23 **B** true absolute partial off-diagonal correlations. As the number of edges increases, the true correlation values decrease to maintain the positive definite property of the partial correlation matrix. Consequently, as the number of edges increases, AUC-ROC performance declines

**Fig. 24:**
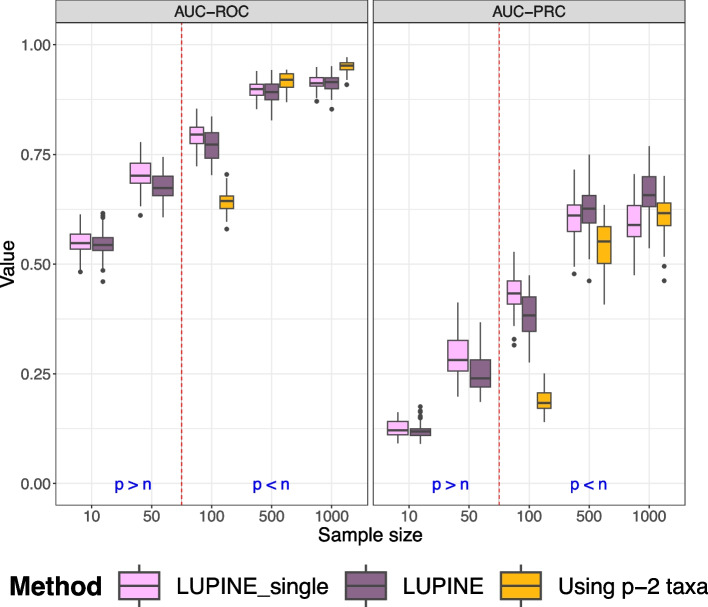
Simulated data. Box plots illustrating the Area Under the Receiver Operating Characteristic (AUC-ROC) and Precision-Recall Curve (AUC-PRC) for LUPINE_single, LUPINE, and a comparative method using all $p-2$ taxa for partial correlation estimation when $p<n$. The edge proportion is set at 0.1. Performance improves for all methods as sample size increases. At sample size of 100, LUPINE methods outperform the comparative approach in both AUC-ROC and AUC-PRC measures. This advantage decreases with larger sample sizes, especially for AUC-ROC

## Data Availability

No datasets were generated. All the data analysed in the manuscript are publicly available on Github https://github.com/SarithaKodikara/LUPINE_manuscript.

## Appendix A: Assessing the efficacy of the approximation used to improve the computation time

In this section, we evaluate the adequacy of the approximation used to improve computatoinal time using the normal diet data on day 0 from the HFHS case study ($X_0$). As a reminder, our method involves the computation of one-dimensional approximations for $p-2$ taxa. In the simplest case of a single time point, we achieved this by systematically excluding pairs of taxa from the initial dataset $X_0$, performing a principal component analysis (PCA) and extracting the first principal component. Given that there are $p \times (p-1) /2$ possible ways of selecting the excluding taxa pair, we performed PCA $p \times (p-1) /2$ on $X_0^{-(i,j)}$. $X_0^{-(i,j)}$ denote the data set $X_0$ after excluding the *i*th and *j*th taxa.

To improve computational efficiency and avoid repetitive PCA computation, we employed an approximation strategy for calculating the first principal component of $X_0^{-(i,j)}$. We first compute the loading vector for the entire dataset, $X_0$, then, approximate the first principal components for $p-2$ taxa by selectively nullifying the loading weights corresponding to the excluded taxa pair, then multiplying the resulting loading vector by $X_0$.

To compare the original principal component with its approximation we used the concordance correlation coefficient. The concordance correlation coefficient evaluates the similarity between paired data by quantifying the deviation from the concordance line ($45^{\circ }$ line through the origin) [66]. A concordance correlation value close to 1 signifies that when plotting the original principal component against its approximated counterpart, the values closely adhere to the concordance line, thus indicating a high level of agreement between the original and the approximated principal component. In Fig. 13, we observe that all the concordance correlation values are above 0.99 between the approximated and the original principal component indicating a high level of agreement between the two. In the remainder of the main article, we therefore chose to use approximated principal component to calculate the partial correlations.

## Appendix B: Simulation design

In this section, we describe the details of the simulation study for comparing network inference methods.

We generate data using a multivariate Poisson distribution and incorporate concepts inspired by birth and death processes [67]. At the initial time point, we use a copula-based multivariate Poisson distribution to generate 54 taxa counts for each individual. For each subsequent time point, we simulate the occurrence of new births using a multivariate Poisson process. We then update the taxa counts by adding these new births to the previous counts, which are reduced to simulate the concept of ‘death’. To simulate ‘death’, we incorporate the First-order Integer-valued Autoregressive (INAR(1)) mechanism. This mechanism employs a binomial thinning operator to model non-negative integer-valued time series [68]. This operator represents the decrease in taxa population between two time points. To understand the idea of a binomial thinning operator, consider a population of size *Y* at a specific time *t*. If we observe the same population at time $t + 1$, the population may decrease due to the death of some entities between times *t* and $t + 1$. Assuming these deaths are independent events and that the probability of dying between *t* and $t + 1$ is uniform, denoted by $1-\alpha$, then the resulting count of survivors at $t + 1$ can be expressed as $\alpha \circ Y$ [69]. Thus, at a given time point *t*, taxa count matrix, $C_t$, is modelled as

14 $$\begin{aligned} C_{t} = \alpha \circ C_{t-1}+ N_t \end{aligned}$$

where $C_{t-1}$ represents the taxa count matrix at time $t-1$; $\alpha$ is the survival rate; and $N_t$ is the new birth matrix at time *t*. The matrices $C_{t}$, $C_{t-1}$, and $N_t$ correspond to *n* individuals and 54 taxa.

To preserve the correlation structure, we use a correlated binomial distribution for thinning, with $\alpha = 0.5$ until time point 6 and $\alpha = 0.1$ thereafter. We assume the expected value of each taxon *i* for individual *j* ($\lambda _{ij}$) in the multivariate Poisson distribution remains constant over the two time periods: days 1–5 and days 6–10. We generate these $\lambda _{ij}$ values using two different Gamma distributions. For the first period (days 1–5), we generate $\lambda _{ij}$ using a Gamma distribution with its parameters estimated from the mean taxon values in the normal diet group. For the second period (days 6–10), we generate another $\lambda _{ij}$ using a different Gamma distribution with its parameters estimated from the mean taxon values in the HFHS diet group. To assess the goodness of fit between the observed mean taxon values and the fitted Gamma distribution, we generate quantile-quantile (Q-Q) plots (Figs. 14 and 15). These Q-Q plots confirm the goodness of fit for the majority of taxa, as evidenced by the close alignment between the points and the theoretically expected values of the distribution.

Finally, to mimic the library size effect in our simulation, we rarefy the samples to be between 5000 and 8000.

## Appendix D: Sensitivity analysis

In this section, we performed a sensitivity analysis to assess the robustness of LUPINE and LUPINE_single across various conditions using simulated data. The key factors evaluated include the choice of statistical test (correlation test vs. permutation-based test), normalisation techniques, number of components used in deflation, dimensionality reduction methods, edge proportions, and sample size. Our results showed that:

- Correlation-based inference were more efficient than permutation-based methods, specifically for large sample size.
- Normalisation methods showed no significant impact on model performance.
- Using one component for deflation yielded comparable or superior performance to using multiple components, especially for smaller sample sizes.
- Alternative dimension reduction methods to PCA, such as robust PCA (RPCA) and independent component analysis (ICA) produced similar outcomes, with PCA showing slight advantages for larger sample sizes.
- Model performance decreased as edge proportions increased, though AUC-PRC results suggested that significant edges still captured true correlations.
- As expected, larger sample sizes enhanced model performance due to improved signal detection.

These findings reinforce the stability of LUPINE and LUPINE_single under various configurations.

### Permutation test vs. correlation test

In LUPINE, we proposed using a correlation test to infer associations between taxa. On simulated data, we compared the association inferences from a permutation-based test as follows. We first calculating the partial correlation between taxa *i* and *j*, as shown in Eq. 5 (referred to as the original correlation). Next, we permuted the residuals from taxon *j* ($e_j$) to eliminate any association between *i* and *j* and then calculated the correlation between $e_i$ and the permuted $e_j$. This procedure was repeated 100 times, after which we calculated the proportion of instances where the absolute value of the original correlation was less than the absolute value of the permuted correlation. A lower proportion indicated a significant association. We compared this permutation method with our correlation test results on simulated data with $n=23$ and $n=120$ in the ‘Simulation study’ section. As seen in Fig. 19, correlation-based inference identified the true correlations more accurately than the permutation-based inference approach, particularly when considering the PRC values. The computational time for the permutation-based method increased exponentially with the number of nodes (i.e. taxa) as we need to perform permutations for each pair of taxa.

### Effect of normalisation

In our original LUPINE approach, we applied PCA to the count matrix, with taxa counts centred and scaled. For the linear regressions involving taxa *i* and *j*, we used the PCs as explanatory variables, and log-transformed the taxa counts from *i* and *j* to convert them to a continuous scale before using them as a response variable.

In this section, using simulated data, we evaluated the sensitivity of the results when PCA was performed on the log or clr transformed counts. Thus, in this subsection, we compared the sensitivity of LUPINE_single and LUPINE on simulated data with $n=23$ and $n=120$ (as described in the ‘Simulation study’ section) using the following three approaches:

1. (Default) In the linear regressions involving taxa *i* and *j*, the explanatory variables are the log library size and the first principal component from the centred and scaled counts (excluding taxa *i* and *j*), while the response variables are the log-transformed counts of taxa *i* and *j*.
2. As in 1, but count data are log-transformed in PCA.
3. Explanatory variable is the first principal component from the centred and scaled clr transformed counts (excluding taxa *i* and *j*) and the response variables are the clr transformed counts of taxa *i* and *j*.

In the simulation, we did not observe significant differences in the three approaches considered (Fig. 20). Thus, in situations where count data are available, we recommend using our default version. However, users of LUPINE can choose between count or log/clr transformed data.

### Effect of using different number of components in the deflation

In our original approach, we used one component in the deflation to account for the largest variation in the control taxa. Including more components, however, could potentially improve model performance. To assess this, we evaluated model performance using one to three components. Our simulation results showed that using just one component produced similar or better outcomes compared to using two or three components, across two sample sizes (Fig. 21). In particular, the one-component approach performed better when the sample size was small.

### Effect of using different dimension reduction methods in LUPINE_single

In LUPINE_single, we used PCA as the dimensionality reduction method. Armstrong et al. [70] suggested other dimension reduction methods that might be suitable for microbiome data. We focused on RPCA, as it was available in R and could be easily integrated into our package. We also considered ICA. We obtained similar performance for PCA, ICA, and RPCA on our simulated data. However, PCA showed slightly better performance with a larger sample size ($n = 120$) (Fig. 22). Thus, PCA is kept as the default option in the package, with alternatives for RPCA and ICA.

### Effect of number of edges

To evaluate the performance of the LUPINE_single and LUPINE models, we tested them using different edge proportions with a sample size of 23. Edge proportion was defined as the ratio of observed edges to the total number of possible edges. Model performance, measured by AUC-ROC, decreased as the edge proportion increased from 0.005 to 0.8 (Fig. 23A). This decline occurred because increasing the number of edges reduced the true partial correlations. Maintaining the positive definiteness of the partial correlation matrix required this adjustment (Fig. 23B). As partial correlations decreased, detecting significant correlations that differed from zero became more difficult, resulting in lower performance. However, performance based on AUC-PRC initially decreased and then improved, suggesting that significant edges were more likely to represent true correlations.

### Effect of sample size

We tested model performance across different sample sizes using an edge proportion of 0.1. The choice of a 0.1 edge proportion was inspired by the edge proportions in the simulated network shown in Fig. 4A, which was inferred using SpiecEasi. Additionally, performance was generally worse at this edge proportion, as seen in the AUC-ROC and AUC-PRC values in Fig. 23.

LUPINE_single and LUPINE use a one-dimensional approximation of $p-2$ taxa when calculating partial correlations. However, when $p<n$, it is also possible to estimate partial correlations without this approximation: we use all $p-2$ controlled taxa in the regressions in Eq. 3. As n approaches infinity, using all $p-2$ taxa should converge to the actual partial correlation. Therefore, in this section, we evaluated LUPINE_single and LUPINE alongside a new approach that calculates partial correlations using all $p-2$ taxa when $p<n$.

Increasing the sample size improved the signal-to-noise ratio, enhancing the performance of all methods (Fig. 24). For a sample size of 100, nearly double the number of features, the LUPINE approaches outperformed the method using $p-2$ taxa in both AUC-ROC and AUC-PRC measures. However, as the sample size increased, the performance difference between the methods decreased, specifically for AUC-ROC values.
